# A human fetal lung cell atlas uncovers proximal-distal gradients of differentiation and key regulators of epithelial fates

**DOI:** 10.1016/j.cell.2022.11.005

**Published:** 2022-12-08

**Authors:** Peng He, Kyungtae Lim, Dawei Sun, Jan Patrick Pett, Quitz Jeng, Krzysztof Polanski, Ziqi Dong, Liam Bolt, Laura Richardson, Lira Mamanova, Monika Dabrowska, Anna Wilbrey-Clark, Elo Madissoon, Zewen Kelvin Tuong, Emma Dann, Chenqu Suo, Isaac Goh, Masahiro Yoshida, Marko Z. Nikolić, Sam M. Janes, Xiaoling He, Roger A. Barker, Sarah A. Teichmann, John C. Marioni, Kerstin B. Meyer, Emma L. Rawlins

**Affiliations:** 1 Wellcome Sanger Institute, Hinxton, Cambridge CB10 1SA, UK; 2 European Molecular Biology Laboratory, European Bioinformatics Institute (EMBL-EBI), Wellcome Genome Campus, Cambridge, UK; 3 Wellcome Trust/CRUK Gurdon Institute, Department of Physiology, Development and Neuroscience, University of Cambridge, Cambridge CB2 1QN, UK; 4 Molecular Immunity Unit, University of Cambridge Department of Medicine, Cambridge, UK; 5 Department of Paediatrics, Cambridge University Hospitals, Hills Road, Cambridge CB2 0 QQ, UK; 6 Biosciences Institute, Newcastle University, Newcastle upon Tyne, NE2 4HH, UK; 7 Lungs for Living Research Centre, UCL Respiratory, University College London, London, UK; 8 John van Geest Centre for Brain Repair, Department of Clinical Neurosciences and Wellcome-MRC Cambridge Stem Cell Institute, University of Cambridge, Cambridge, UK; 9 Department of Physics, Cavendish Laboratory, University of Cambridge, Cambridge CB3 0HE, UK; 10 Cancer Research UK Cambridge Institute, University of Cambridge, Cambridge, UK

## Abstract

We present a multiomic cell atlas of human lung development that combines single-cell RNA and ATAC sequencing, high-throughput spatial transcriptomics, and single-cell imaging. Coupling single-cell methods with spatial analysis has allowed a comprehensive cellular survey of the epithelial, mesenchymal, endothelial, and erythrocyte/leukocyte compartments from 5–22 post-conception weeks. We identify previously uncharacterized cell states in all compartments. These include developmental-specific secretory progenitors and a subtype of neuroendocrine cell related to human small cell lung cancer. Our datasets are available through our web interface (https://lungcellatlas.org). To illustrate its general utility, we use our cell atlas to generate predictions about cell-cell signaling and transcription factor hierarchies which we rigorously test using organoid models.

## Graphical abstract

**Graphic abstract:**
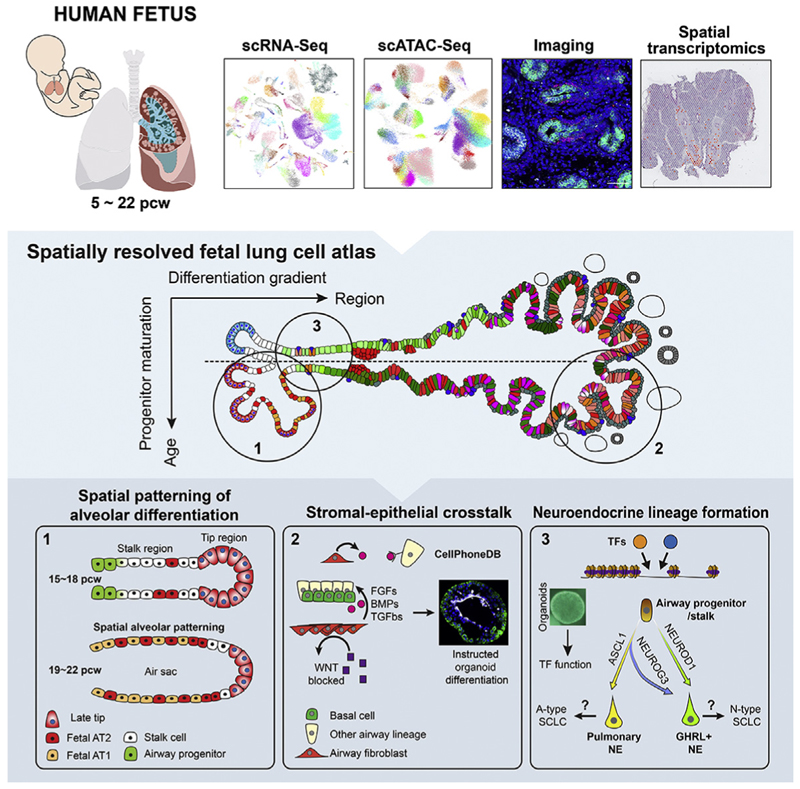

## Introduction

Single-cell mapping of cell states in the adult human lung in health and disease is being performed at increasing resolution,^1^ providing a foundation for understanding lung cellular physiology. The adult lung has low rates of cell turnover,^2,3^ making it difficult to capture transition states and progenitors. Moreover, there are developmental-specific cell states that do not exist in the adult. A high-resolution cell atlas of the embryonic and fetal human lung will identify developmental precursors and progenitors and predict differentiation trajectories and potential gene regulatory networks. This will provide a baseline for studying adult homeostasis and disease.

The lung buds are specified in the human foregut endoderm at ^4,5^ ~5 post-conception weeks (pcw). Subsequent morphogenesis is driven by branching of the distal-most bud tips. The bud tip epithelium comprises SOX9^+^, ID2^+^ multipotent progenitors that self-renew during branching.^6–9^ As the bud tip epithelium branches into the surrounding mesoderm, the epithelial cells that remain in the stalk region start to differentiate into bronchiolar (airway, ~5–16 pcw) and later (from ~16 pcw) into alveolar epithelium^5^. The pattern of growth from multipotent epithelial progenitors at the distal tips means that the position of a cell along the proximal-distal axis of the lung epithelial tree is a strong predictor of its maturity. The more mature cells, which exited the tip first, are more proximal, whereas the most immature cell states, which exited the tip recently, are found in the tip-adjacent (stalk) regions.^10^ In other words, space reflects time in lung development. Therefore, coupling single-cell state analysis to *in vivo* spatial visualization can provide high confidence in the identification of novel progenitor cells in the developing lung. Moreover, detailed spatial analysis of cell states allows cell identity designations to be compared to more traditional histological definitions.

We have generated a high-resolution single-cell atlas of human lung development using a combination of scRNA-seq, scATAC-seq, Visium Spatial Transcriptomics, and mRNA *in situ* hybridization using hybridization chain reaction (HCR).^11^ Combining these data sources has allowed us to identify 144 cell states/types in 5–22 pcw lung samples. These include previously uncharacterized progenitor cell states, transition populations, and a subtype of neuroendocrine cell related to a subtype of human small cell lung cancer (SCLC). We observe increasing cell maturation over time, with many cell states identified in adult lungs already present at 22 pcw. We have used our atlas to make predictions about progenitor cell states, signaling interactions, and lineage-defining transcription factors, and we demonstrate how these can be efficiently tested using a genetically tractable human fetal lung organoid model. The datasets are available for interactive analysis at https://lungcellatlas.org.

## Results

### A single-cell atlas of human lung development comprising 144 cell states

**Figure 1:**
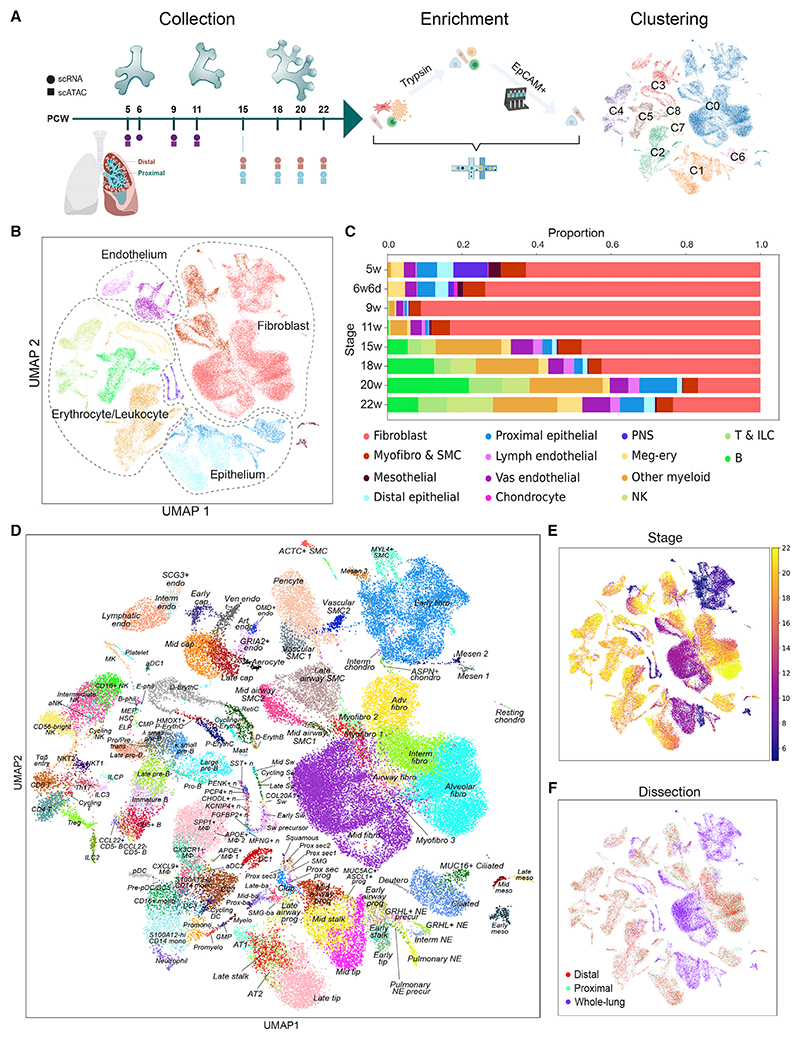
Data and experimental overview (A) Overview of sample collection for scRNA-seq (circles) and scATAC-seq (squares) experiments from whole lung (purple), distal (red), and proximal (blue) regions, cell processing and broad clustering; cluster number refers to the data portal (https://lungcellatlas.org). (B and C) UMAP representation (B) and cell-type proportion (C) of 71,752 good-quality cells, indicating epithelial, endothelial, fibroblast, and leukocyte/erythroid compartments. (D–F) UMAP visualization by cell type/state (D), developmental stage (E), and dissection region (F). See also Figures S1 and S2.

We obtained human embryonic and fetal lungs from 5–22 pcw for scRNA-seq and scATAC-seq. To focus on differentiation, we deeply sampled 15, 18, 20, and 22 pcw lungs and separated proximal and distal regions, while leaving lungs at 5, 6, 9, and 11 pcw intact. We used a mixture of cell dissociation methods to obtain a balanced mixture of cell types (Figure 1A) and produced highquality transcriptome (Figure S1A; average > 2,400 genes/cell) and DNA accessibility (Figures S1K and S1L; average > 18,000 fragments/nucleus) data. After iterative clustering (Figures S1C and S1D), removal of doublet-driven clusters (Figures S1E, S1G, S1G’, and S1G”), stressed or low-quality clusters (except those expressing known markers, such as erythroid) (Figures S1I, S1I’, and S1I”), clusters composed of cells from only one sample when replicates are available, and clusters of cells from other organs (Figure S1H)^12,13^ and maternal cell evaluation (Figures S1F and S1J), we present 71,752 cells shown as a uniform manifold approximation and projection (UMAP) (Figure 1A), on which we manually annotated fibroblast, epithelial, endothelial, and erythrocyte/leukocyte lineages (Figure 1B). Plotting the cell-type distribution against time (excluding trypsin/CD326-treated samples, shown in Figure S1B) showed that fibroblasts were the most prominent cell, particularly in younger lungs (Figure 1C). Leukocytes and erythrocytes were observed in all lungs sampled, with B, T, and NK cells becoming prominent from 15 pcw (Figure 1C).

Further cell-type annotation was performed based on marker genes (Table S1), resulting in assignment of 144 cell types/states (Figure 1D). Sample age was a strong determinant of clustering (χ^2^ = 163,727, p ≈ 0), reflecting progressive cell maturity over time (Figure 1E). Clusters mostly grouped into three distinct regions which we categorized as early (5, 6 pcw), mid (9–11 pcw), and late (15–22 pcw) stages. Cell cycle phase (Figure S1M, χ^2^ = 25,361, p ≈ 0) and dissected region (Figure 1F, χ^2^ = 968, p = 8.9E-131) were also associated with clustering. However, the dissection region was only prominent for a small number of proximally located cell types (Figure 1F), suggesting that most proximal-to-distal regions of the airway structure were still represented in both dissected regions of the lung. Epithelial cells were mostly derived from the trypsin-treated and CD326-enriched samples, although airway smooth muscle, myofibroblasts, and alveolar fibroblasts were also enriched here (Figure S1M’). Peripheral nervous system (PNS) cells and chondrocytes were only obtained from 5–6 pcw lungs, likely correlating with lower extracellular matrix (ECM) complexity in younger lungs and/or increased fragility of older neurons. PNS cells were clustered and assigned to cell types, but scarcity precluded further analysis (Figure 1D, S1N, and S1N’). Data integration and logistic regression-based comparison showed that gene expression of our annotated cells corresponds well to those of adult lungs^14^ (Figures S2A–S2C).

### a differentiation trajectory of airway progenitor states lies along the developing lung distal-to-proximal axis

**Figure 2:**
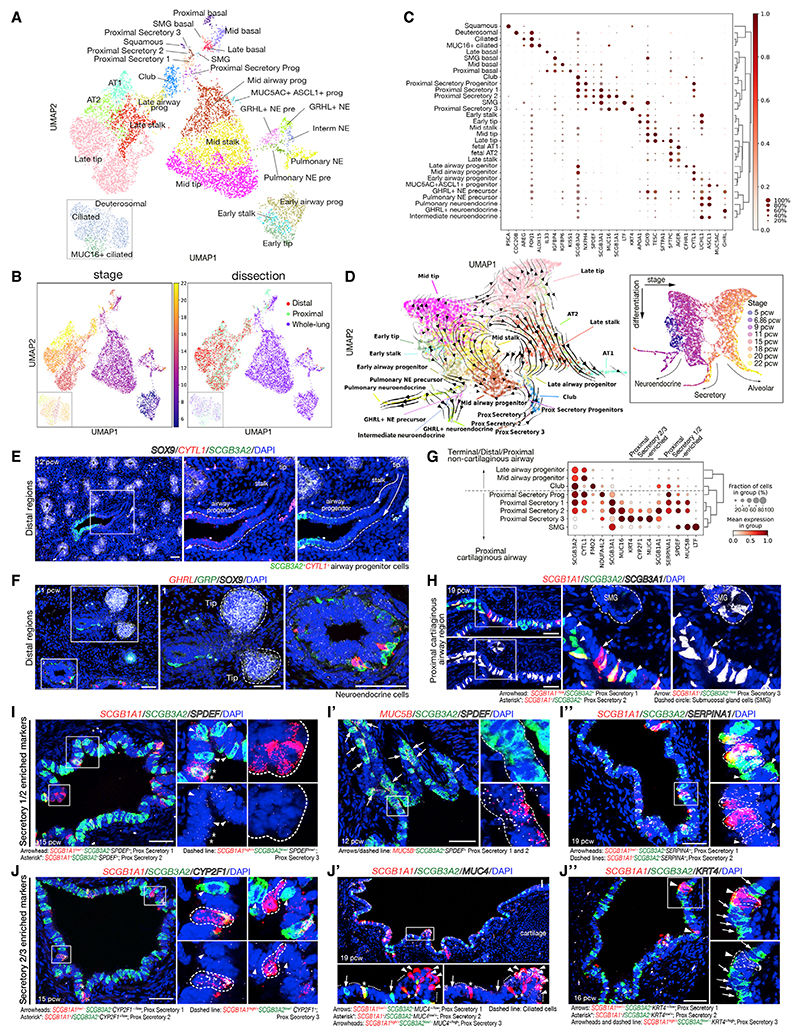
Epithelial cell types, states, and locations over developmental time (A and B) UMAP visualization of epithelial cells, colored by cell types (A), stage (B, left), and region (B, right). (C) Dot plot describing differential marker gene expression level for epithelial cells. (D) UMAP visualizing the predicted epithelial cell lineage trajectory using scvelo; inset: developmental age. (E and F) *In situ* HCR at 11 (F) and 12 (E) pcw. (E) *SOX9* (tip epithelium, white), *CYTL1* (red), *SCGB3A2* (green). (F) *GHRL*^+^ (*GHRL*^+^ neuroendocrine, red), *GRP*^+^ (pulmonary neuroendocrine, green). (G) Dot plot showing differential marker genes across secretory cell subtypes. (H) *In situ* HCR at 19 pcw using *SCGB1A1* (red), *SCGB3A2* (green), and *SCGB3A1* (white). (I and J) Differentially enriched genes in the proximal secretory cell subtypes. *SPDEF* (I, I’), *SERPINA1* (I”), *CYP2F1* (J), *MUC4* (J’), and *KRT4* (J”) all white; *MUC5B* (I’) and *SCGB1A1* all red, and *SCGB3A2* green. DAPI, nuclei. Scale bars, 50 μm. See also Figures S3, S4, and S5.

The epithelial cells separate by age (Figures 2A and 2B), with many basal cells, MUC16^+^ ciliated cells, and secretory cells enriched in the proximally dissected tissue (Figures 2B and S1O). The most immature epithelial progenitors are tip cells: SOX9^+^ multipotent progenitors located at the distal branching tips of the respiratory tree.^8^ Tip cells were separated into early (5,6 pcw), mid (9–11 pcw) and late (15–22 pcw) populations (Figures 2A and 2B) with both shared and stage-specific markers (Figure 2C). On the epithelial UMAP, each tip population clusters closely with adjacent stalk cells (*SOX9*^*LO/-*^, *PDPN*^*LO*^, *HOPX*^*LO*^) and airway progenitors (*CYTL1*^*LO/+*^, *PCP4^+^*, *SCGB3A*^*+/LO*^) (Figure 2A). The tip, stalk, and airway progenitors can be visualized in a distal-proximal sequence in the tissue at all stages tested (10–16 pcw) (Figures 2E, S3A, and S1B, Video S1), consistent with the most proximal cells being the most mature. These three cell types form a predicted differentiation trajectory from mid-tip to mid-stalk to mid-airway progenitor that branches into the neuroendocrine, or secretory, lineages (Figure 2D).

### Two subtypes of neuroendocrine cells are present in the developing airways

Consistent with previous data,^15^ the earliest differentiated epithelial cells detected were neuroendocrine (NE) cells in 5 pcw lungs (Figures 2A–2C). We identified two types of NE cells: classical pulmonary NE cells (*GRP^+^*) and GHRL^+^ NE cells (*TTR^+^*, *GHRL^+^*) in agreement with a recent human fetal cell atlas.^13^ We observed increasing maturity of NE cells over time (specific populations denoted as precursors on the UMAP). In addition, an intermediate NE population, a putative transition state, connected the two NE cells (Figure 2A). At 11 pcw, *GRP^+^* pulmonary NE cells were observed closer to the budding tips, suggesting that they begin to form prior to the *GHRL^+^* NE cells (Figure 2F). This spatial difference was not apparent in the oldest samples where both *GRP^+^* and *GHRL^+^* cells were observed at all airway levels, although less abundant distally (Figure S3C). Mouse *Ghrl^+^* NE cells were not detected in re-analysis of published mouse data,^16,17^ or spatially.^18^ However, *Ghrl* is expressed in mouse ciliated cells that cluster with human fetal GHRL^+^ NE cells (Figure S2D).^17^

### Multiple secretory cell subtypes in the proximal cartilaginous airways

We annotated 5 sub-types of differentiating secretory cells and one proximal secretory progenitor. (1) The proximal secretory progenitors (*SCGB3A2^+^*, *SCGB1A1^-^*, *SCGB3A1*^*-/LO*^, *CYTL1^+^*) were detected in the single-cell atlas at 9 pcw, prominent at 11 pcw, but rarer in older lungs consistent with a progenitor state (Figures 2A–2C, and 2G). (2) Club cells (S*CGB3A2^+^*, *SCGB1A1^+^*, *SCGB3A1^-^*, *SPDEF^-^*, *MUC16^-^*) were detected from 15 pcw in the single-cell data (Figures 2A–2C, and 2G), or 12 pcw in the tissue localized in clusters more distally, but dispersed in the more proximal non-cartilaginous regions (Figure S3D). (3) Submucosal gland (SMG) secretory cells (*LTF^+^*, *SCGB3A1^+^*, *SPDEF^+^*) were detected from 15 pcw in the single-cell data, located in SMG ducts and likely to be a precursor of serous and/or mucoussecreting SMG cells (Figures 2A–2C, 2G, and S3G). (4) Proximal secretory 1 (*SCGB1A1*^*LO*^, *SCGB3A2^+^*, *SCGB3A1^+^*) and (5) proximal secretory 2 (*SCGB1A1^+^*, *SCGB3A2^+^*, *SCGB3A1^+^*) appeared from 11 pcw (Figure 2A–2C, 2G–2H, and S3E). Both were *SPDEF^+^*, *MUC5B^+^*, *SERPINA1^+^* (Figure 2I), suggesting they differentiate into goblet or mucous cells. By contrast, (6) proximal secretory 3 (*SCGB1A1^+^*, *SCGB3A2*^*LO/-*^, *SCGB3A1^+^*) was detected from 15 pcw and was *SPDEF^-^* (Figures 2A–2C), but *CYP2F1^+^*, *MUC4^+^*, and *KRT4^+^* (Figures 2G and 2J). All three luminal proximal secretory cell populations were located in the proximal cartilaginous airways and were *MUC16^+^* (Figures 2C, 2G, 2H, S3E, and S3F). Detailed spatial-temporal analysis of 10–21 pcw airways revealed that the proportion of proximal secretory progenitors decreased with developmental age, while proximal secretory cells 1 and 2 increased (Figures S4A–S4C), consistent with a progenitor function for proximal secretory progenitors.

### Other airway cells

We detected ciliated cells (*FOXJ1^+^*, *ALOX15*^+^) from 11 pcw, interspersed with secretory/club cells (Figures 2A–2C, S3H, S4A, and S4B). Rarer deuterosomal cells (*FOXJ1^+^*, *CDC20B^+^*) appeared at the same time (Figures 2A–2C). MUC16^+^ ciliated cells (*FOXJ1^+^*, *DNAH^+^*, *MUC16*^*LO*^) were also detected from 11 pcw but confined to proximal dissected regions (Figures 2A–2C and S3H). They were located in patches in the most proximal cartilaginous airways (Figure S3I) and likely represent *MUC16^+^* secretory cells generating ciliated cells, as suggested in the adult.^19–21^ Basal cells (*TP63^+^*, *F3^+^*) were present from 9 pcw (Figures 2A–2C and S3J) and more frequent in proximal regions (Figures 2C, S4A, and S4B). Rarer cells (ionocytes, tuft) that have been identified in adult airways were not present in our single-cell data. However, we found putative ionocytes (*FOXI1^+^*; 4/4 lungs) and putative tuft cells (*POU2F3^+^*; 2/4 lungs) in the most proximal cartilaginous airways of 21–22 pcw lung sections (Figure S4E), suggesting they begin to differentiate mid-gestation. Moreover, we reproducibly detected a small population of *MUC5AC*^+^, *ASCL1*^+^ cells in 9–11 pcw lungs (Figures 2A–2C). These were localized to the proximal non-cartilaginous airways where they appeared as solitary, somewhat basal, non-columnar cells (Figure S3K). We hypothesize that they are an unknown progenitor, consistent with their transient appearance and the observation that Ascl1^+^ NE cells in adult mice can generate club, ciliated, and mucous cells following injury.^22,23^

### Predicted airway epithelial differentiation trajectories

A detailed spatiotemporal analysis of major airway epithelial cell types from 10–21 pcw confirms that cell maturation begins more proximally. An example is lack of ciliated and club cells in the distal non-cartilaginous airways at 10–12 pcw but presence at 15–21 pcw (Figures S4A–S4D). Conversely, airway progenitors are found throughout the non-cartilaginous airways at 10–12 pcw but restricted to terminal airways by 15–21 pcw (Figures S4A–S4D). In addition, proximal secretory cells are spatially restricted to the cartilaginous airways, while club cells are found in the non-cartilaginous regions (Figures S4A–S4D).

This spatial separation means that predicted differentiation trajectories that combine proximal secretory cells and club cells (Figure 2,D) can reveal general trends but are likely to be oversimplified. We therefore predicted mid- (Figures S5A–S5C) and late-stage (Figures S5D–S5F) airway lineage trajectories separately. In both cases, basal cells formed discrete clusters on the UMAPs (Figures S5A’ and S5D’). Trajectory inference analysis suggests a differentiation route from mid-tip to stalk to airway progenitors to proximal secretory progenitors and proximal secretory cells (Figure S5B), consistent with sample age (Figure S5B’). Visualizing gene expression along the inferred trajectory shows mid-tip and stalk cells are similar (Figure S5C). Stalk cells lose some tip markers, including *FOXP2* and *SOX9*, and gain a small number of genes, including *PDPN* and *AGER*. By contrast, the newly defined airway progenitors upregulate marker genes associated with airway fates, including *CYTL1, CLDN4*, and *SCGB3A2*^24,25^ (Figure S5C). A similar differentiation trajectory was predicted from late-tip to late-stalk to late-airway progenitor to club cells (Figure S5E), although the oldest tip and stalk cells included in this analysis may produce alveolar lineages (Figures S5E’, 3C, 3E, S6A, and S6B). Visualizing gene expression along the inferred late-airway trajectory shows that the late-tip and stalk cells are transcriptionally similar and undergo gene expression changes analogous to mid-tip and stalk (loss of *SOX9, FOXP2*; gain of *PDPN, AQP5*; Figure S5F).

These analyses predict that cells exit the tip to the stalk state, followed by gain of airway progenitor identity before commitment to a specific differentiation state that likely depends on local signaling cues. Although we cannot predict the origin of the basal cells using trajectory inference methods, we hypothesize that they are derived from a columnar progenitor (possibly the airway progenitor) but will themselves act as progenitor/stem cells following differentiation analogous to previous observations in mice.^26^

Our trajectory inference (Figures S5A–S5F) predicts that airway progenitors will differentiate readily to airway cell types. At 9–10 pcw, *CYTL1^+^* and *SCGB3A2^+^* airway progenitors are found throughout the airway tree (Figures 2E, S3B, S3D, S3L, S4A, S4B, and S4D). We isolated airway progenitors using a combination of distal non-cartilaginous airway micro-dissection and transduction with a lentiviral *SCGB3A2* transcriptional reporter (*SCGB3A2-GFP*, Figure S5G). Freshly isolated distal *SCGB3A2*-GFP^+^ cells were *SOX9*^*LO*^, *CYTL1*^*HI*^, *SCGB1A1*^*LO*^, *SCGB3A2*^*LO*^, and *SCGB3A1*^*LO*^ compared to tip/stalk cells and more proximal *SCGB3A2*-GFP^+^ cells from the same lungs (Figure S5H). When single cells were placed into an FGF-containing differentiation medium,^27^ distal *SCGB3A2*-GFP^+^ cells produced basal, ciliated, and mature secretory cells (Figures S5I–S5M). This demonstrates that, consistent with the trajectory analysis, the airway progenitors are competent to differentiate into airway lineages.

In summary, we have identified multiple epithelial progenitor states (tip, stalk, airway progenitor, and proximal secretory progenitor) and differentiating airway cells that localize to a spatial differentiation gradient along the proximal-distal axis of the epithelium (summarized in Figures S3L and S4D). Moreover, we identify *GHRL^+^* neuroendocrine cells that do not exist in the mouse.

### Late epithelial tip cells acquire alveolar identity prior to alveolar differentiation

**Figure 3:**
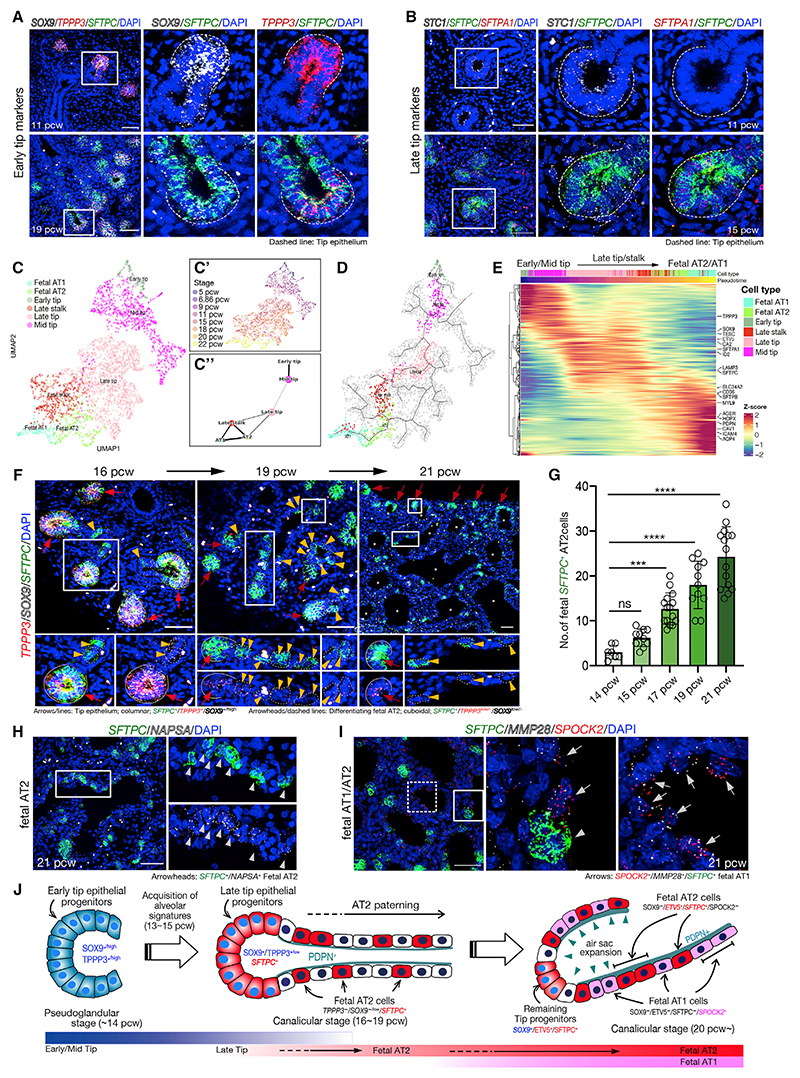
Late epithelial tip cells acquire an alveolar progenitor identity (A and B) *In situ* HCR at 11 (A and B), 15 (B), and 19 (A) pcw. (A) *SFTPC* (green), *TPPP3* (red), *SOX9* (white). (B) *SFTPC* (green), *SFTPA1* (red), *STC1* (white). Dashed lines represent tip epithelium. (C and D) UMAP visualization of early to late tip, late stalk, fetal AT1 and AT2 cells, colored by cell types (C) and stages (C’); PAGA analysis (C”); Monocle3 trajectories (D). (D) Gene expression heatmap of trajectory colored in (D). (E) *In situ* HCR at 16, 19, and 21 pcw, *SFTPC* (green), *TPPP3* (red), and *SOX9* (white). White lines/red arrows: columnar tip progenitors, *SFTPC*^+^/*SOX9*^+/high^/ *TPPP3*^+^. Arrowheads/dashed lines in stalk/air sac regions: cuboidal differentiating fetal AT2 cells, *SFTPC*^+^/*SOX9*^low/-^/*TPPP3*^low/-^. Asterisks (*) represent primitive air sacs. (F) Quantification of cuboidal *SFTPC*^+^/*SOX9*^low/-^ fetal AT2 cells in stalk/air sac regions in (F). The *SFTPC*^+^ tip epithelial cells were excluded by their columnar morphology and marker expression (*SOX9*^low/-^). Mean ± SD, n > 7. Significance evaluated by one-way ANOVA with Tukey multiple comparison post-test; ns: not significant, *p < 0.05, **p < 0.01, ***p < 0.001, ****p < 0.0001. (H and I) *In situ* HCR analysis at 21 pcw. Fetal AT2 *SFTPC*^+^ and *NAPSA*^+^ (arrowheads; H and I) and fetal AT1 *SFTPC*^-^/*MMP28*^+^/SPOCK2^+^ (arrows; I). (J) Diagram of the acquisition of alveolar progenitor identity by late epithelial tips, followed by differentiation to fetal AT2 and AT1 lineages. DAPI, nuclei. Scale bars, 50 μm. See also Figure S6.

Tip cells express a core set of tip-specific markers (*SOX9^+^*, *ETV5^+^*, *TESC^+^*, *TPPP3^+^*, and *STC1^+^*) at all stages sampled (Figures 2A– 2C). We observed a gradual decrease in tip marker expression and an increase in alveolar type 2 (AT2) cell gene expression in tip cells with developmental age (Figure 2C). By 15 pcw the AT2 markers *SFTPC* and *SFTPA* were detected readily in late-tip cells where they were co-expressed with lower levels of core tip markers (Figures 3A and 3B). The late tip is a transcriptional state that has not been detected in developing mouse lungs.^17,28^ The change in expression profile that is observed upon the transition to late tips correlates with a change in the predicted differentiation trajectory from late-tip cells to late-stalk to fetal AT2 and AT1 cells (Figures 3C and 3D; without late stalk in S6A). However, trajectory inference analysis at this transitional stage is challenging. It is likely that some of the late-tip cells produce the terminal branches of the conducting airways (Figures S5D–S5F). Moreover, the inferred connections between mid-tip and late-tip cells are weak (Figure 3C), and we cannot exclude a novel origin for late-tip cells perhaps emerging as new buds from a stalk position, although this hypothesis is not strongly supported by our analysis (Figure 2D). Nevertheless, throughout this period, similar to earlier stages, late-tip cells remain SOX9^+^ and late-stalk cells turn off tip markers and acquire *PDPN/AGER* (Figures S3A and S6D).

A small number of AT2 cells appear in the single-cell data from 15 pcw but are more prominent from 22 pcw (Figure 2A). Similarly, at around 16 pcw, late-tip cells (*SOX9^+^*,*TPPP3^+^*,*SFTPC^+^*) were clearly visualized in the tissue, but differentiating AT2 cells (*SOX9*^*LO/-*^,*TPPP3*^*LO/-*^,*SFTPC^+^*) were rare (Figures 3F and 3G, and S6C). Over the following weeks, the size of the tip regions decreased and more differentiating AT2 cells were detected (Figures 3F and 3G). At 21 pcw, smaller numbers of late-tip cells persist, and AT2 cells (*SOX9^-^*, *SFTPC^+^*, *NASPA^+^*, *ETV5^+^*) were found scattered throughout the developing air sacs (Figures 3H and S6E–S6J). Consistent with the predicted change in tip fate potential (Figures 3C–3E), late-tip cells (16–20 pcw) grown as organoids retained a late-tip phenotype *in vitro* and more readily differentiated to mature AT2 cells than organoids derived from earlier developmental stages.^29^

In our single-cell atlas, differentiating AT1 cells were first visible at 18 pcw but more prominent by 22 pcw (Figures 2A–2C). Similarly in tissue sections, AT1 cells were not detected at 17 pcw (Figure S6H). However, by 20 pcw, differentiating AT1 cells (*SPOCK2*^*LO*^, *SFTPC^-^*) were visible, and at 21 pcw, AT1 cells (*SPOCK2^+^*, *SFTPC^-^*) were interspersed with AT2 cells lining the developing air sacs (Figures 3I, S6I, and S6J). In sections, AT1 markers were only detected in cells which had no or extremely low levels of *SFTPC* (Figures S6H–S6J). Moreover, *SFTPC*-negative cells were always observed in the stalk regions from 16 pcw onwards (Figure 3F). These data are consistent with an alveolar epithelial differentiation model in which, from ~16 pcw, the late-tip progenitors first exit the tip state, turning off AT2 cell markers, and enter the late-stalk cell state, prior to initiating AT1 or AT2 cell differentiation in response to local signaling cues (Figure 3J). Furthermore, the late-stalk cells are connected to AT2, AT1, and late airway progenitors in trajectory inference analysis (Figures 2D, 3C, and 3D), supporting our hypothesis that at all stages of lung development, cells exit the tip and enter a stalk state prior to differentiation.

Integration of our fetal cell atlas with adult data revealed high correlation between expected groups: fetal airway progenitors with adult secretory club cells, fetal and adult ciliated and deuterosomal cells, and proximal secretory fetal cells with adult goblet cells (Figure S2A). The AT2 and AT1 cells we detect in the fetal lungs cluster closely with the adult (Pearson correlation coefficients: fetal-adult AT2 0.66; AT1 0.80). However, the fetal cells are immature and differ in gene expression to their adult counterparts (Figure S2G).

### Lung endothelial cells exhibit early specialization into arterial and venous identities

At 5–6 pcw, the endothelial cells (ECs) comprised capillary (early Cap: *THY1^+^*, *CD24^+^*), GRIA2^+^ arterial (*GRIA2^+^*, *GJA5^+^*), and lymphatic ECs (*PROX1^+^*, *STAB1^+^*, and *UCP2*^*LO*^), showing that capillaries and lymphatic vessels are distinct from the earliest stages of lung development and that arterial specification begins prior to venous (Figure S1T). At later stages, trajectory analysis predicts that both mid- and late-Cap cells generate arterial and venous ECs (Figure S7A). Aerocytes (*CA4*^*LO*^, *S100A3^+^*), capillary ECs specialized for gas exchange and leukocyte trafficking,^30,31^ were observed at 20–22 pcw around the developing air sacs (Figure S7B). Microvasculature specification therefore occurs relatively late in human fetal life coincident with the development of AT1 cells.

Broad markers of arterial and venous specification were clear in sections at 20 pcw (Figures S7C and S7D). Three distinct arterial ECs were detected. GRIA2^+^ and arterial ECs (*DKK2^+^*, *SSUH2^+^*) form a continuous differentiation trajectory in pseudotime (Figure S7A) with GRIA2^+^ ECs likely to be a more immature form. The OMD^+^ ECs (*GJA5^+^*, *DKK2^+^*, *PTGIS^+^*, and *OMD^+^*) cluster with arterial ECs and are more proximal (Figure S1O). By contrast, venous ECs (*PVLAP^+^*, *ACKR3^+^*, and *HDAC9^+^*) do not have clear subclusters. Systemic and pulmonary circulation ECs have been found in adult lungs^32^; we cannot detect these in fetal lungs. Two major lymphatic ECs were detected: lymphatic ECs (*PROX1^+^*, *STAB1^+^*, and *UCP2*^*LO*^) and SCG3^+^ lymphatic ECs (*PROX1^+^*, *SCG3^+^*) (Figure S7E). SCG3^+^ lymphatic ECs resemble a lymphatic valve population.^33^

### Hematopoietic cell types in the developing lung

At the early stages (5–6 pcw) when arterial, capillary, and lymphatic ECs were present, embryonic erythrocytes, HMOX1^+^ erythroblasts, and a small number of macrophages and ILC progenitors were detected, representing the early progenitors of hematopoiesis. After 11 pcw, relative numbers of lymphoid and myeloid cells increased, dominated by macrophages; ILCs; and dendritic, NK, T, and B cells (Figures 1C– 1E, S1P,-S1R, and S1T). Immature T cells are largely absent from the atlas, consistent with the restriction of T cell development to the thymus. In contrast, a range of early B cell precursors and the ILC precursor were detected. TCR and BCR scRNA-seq supported cell-type identities and subdivision (Figures S1Q” and S1R”). We compared our atlas with a pan-fetal human atlas^13^ and found that leukocytes were transcriptionally highly similar to those of other organs (Figure S1S).

### Developmental trajectories of mesenchymal cells

**Figure 4:**
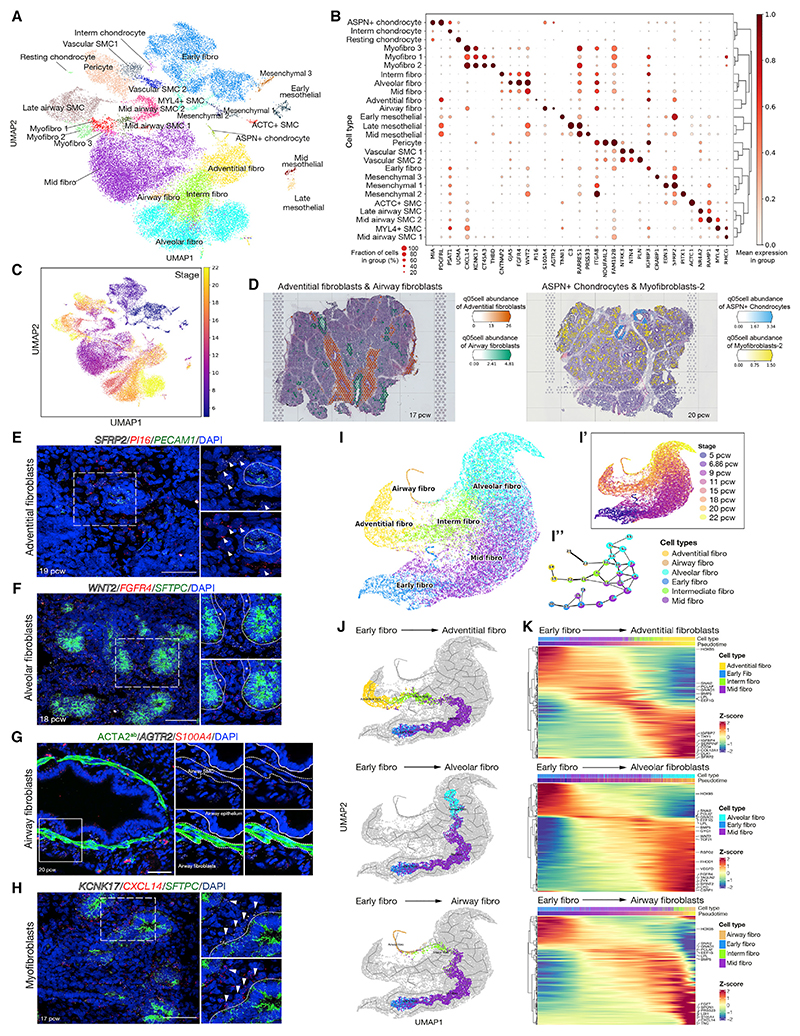
Diverse mesenchymal cell types localize to distinct niches in the developing human lung (A) UMAP visualization of mesenchymal cells. (B) Dot plot of mesenchymal differential marker gene expression. (C) UMAP visualization of mesenchymal cells colored by stage. (D) Visium spatial feature plots visualizing adventitial fibroblasts, airway fibroblasts, ASPN^+^ chondrocytes, and myofibroblast-2 on 17 and 20 pcw lung sections. Scores are conservative estimates of cell-type abundance per voxel. (E–H) *In situ* HCR assay (E–H) and immunostaining (G). (E) Adventitial fibroblasts (*SFRP2*, white/*PI16*,red; arrowheads), ECs (*PECAM1*, green). (F) Alveolar fibroblasts (*WNT2* white; *FGFR4* red), tip cells (*SFTPC* green). Asterisks (*myofibroblasts). (G) Airway fibroblasts (*S100A4* red; *AGTR2* white), smooth muscle (ACTA2 green, dashed line). (H) Myofibroblasts (*KCNK17* white, *CXCL14* red; arrowheads), tip cells (*SFTPC*, green). DAPI, nuclei. Scale bars, 50 mm. (I) UMAP visualization of cell types (I) and stage (I’) and PAGA analysis (I”) of fibroblast differentiation trajectories. (J and K) UMAPs with Monocle3 trajectories (J) and selected trajectory gene expression heatmaps (K) for mid tip to adventitial fibroblasts (top), alveolar fibroblasts (middle), or airway fibroblasts (bottom). See also Figure S7.

The broad fibroblast cluster comprises fibroblasts, myofibroblasts, airway and vascular smooth muscle (ASM and vSMC), pericytes, mesothelium, and chondrocytes (Figures 4A and 4B). Airway fibroblasts and chondrocytes were proximally enriched and mesothelium distally enriched (Figures 4D and S1O). Cell clusters separated by age (Figure 4C). ASM cells were observed from 9 pcw, consistent with previous immunostaining,^8^ and showed increasing maturity over time (Figures 4A–4C). Two distinct populations of vSMC were observed throughout the time course, vSMC1 (*NTRK3^+^*, *NTN4^+^*, and *PLN^-^*) and vSMC2 (*NTRK3^+^*, *NTN4^+^*, and *PLN^+^*) (Figures 4A and 4B), and were intermingled around the same vessels on tissue sections (Figures S7F and S7H). vSMC1 was enriched in genes relating to ECM organization and cell adhesion, and vSMC2 for transcripts encoding contractility proteins and signaling molecules (Figure S7G). Intermingling of vSMC subtypes with different levels of contractility proteins is seen in adult lungs^34^; our developmental observation suggests that these represent normal functional/ontological differences, rather than pathology. Pericytes (*FAM162B^+^*) were visualized adjacent to the microvascular endothelium (Figure S7I).

The most common cells isolated from 5–15 pcw lungs were fibroblasts (Figure 1C). At 5–6 pcw, early fibroblasts (*SFRP2^+^*, *WNT2^+^*) predominated, although multiple populations were detected (Figures 4A and 4B). In 9–11 pcw lungs, early fibroblasts had matured into mid fibroblasts (*WNT2^+^*, *FGFR4*^*LO*^) which can promote epithelial tip cell fate.^35^ In the oldest lungs sequenced, there were three distinct fibroblasts: adventitial (*SFRP2^+^*, *PI16*^+^), airway (*AGTR2*^+^, *S100A4^+^*), and alveolar (*WNT2^+^*, *FGFR4^+^*) with distinct locations (Figures 4A, 4B, and 4D–4G). In addition, an intermediate fibroblast connected the more mature fibroblasts on the UMAP (Figures 4A and 4B), possibly representing a transitional state. Pseudotime analysis predicted a differentiation hierarchy from the early and mid fibroblasts to adventitial fibroblasts, with alveolar and airway fibroblasts forming separate branches (Figures 4I–4K). Alternatively, the intermediate fibroblast population may indicate lineage plasticity as previously suggested.^36^

The three major fibroblast types in 15–22 pcw lungs expressed high levels of genes associated with ECM organization but had distinct gene expression patterns and spatial localization. Adventitial fibroblasts (*SFRP2^+^*, *PI16^+^*) surrounded the larger blood vessels (Figure 4D). They formed diffuse layers of cells surrounding the tightly packed concentric rings of ECs, pericytes, and vSMCs (Figures 4E and S7H). Adventitial fibroblasts were enriched in genes associated with ECM organization and signaling, including BMP, TGFb, and WNT (Figures 4J, 4K, and S7J), consistent with described roles providing structural support to the perivascular region.^37^ Alveolar fibroblasts (*WNT2^+^*, *FGFR4^+^*) were observed throughout the lung, particularly surrounding tip cells and microvasculature (Figure 4F). They were enriched in genes associated with actin organization, focal adhesions, and morphogenesis, as well as signaling molecules (Figures 4J, 4K, and S7J). Adventitial and alveolar fibroblasts expressed shared and unique genes (adventitial: *SERPINF1, SFRP2*, and *PI16*; alveolar: *FGFR4, VEGFD;* Figure 4K). By contrast, the airway fibroblasts (*AGTR2*^+^, *S100A4^+^*; note *S100A4* is expressed in various immune and airway epithelial cells) were adjacent to the ASM and highly enriched in signaling molecules associated with morphogenesis (Figures 4D, 4G, 4J, 4K, and S7J). We did not detect lipofibroblasts,^38^ meaning that they are either rare, form later than 22 pcw, or do not form distinct clusters in all lung datasets.^14^ Endothelial and fibroblast populations align well between fetal and adult data (Figures S2B and S2C), but with some unique developmental states, such as fetal early/mid-fibroblasts and myofibroblasts.

Myofibroblasts formed three distinct groups in our single-cell data. Myofibroblast 1 (*CXCL14^+^*, *KCNK17^+^*, *CT45A3^+^*, and *THBD*^*LO*^) appeared at 9 pcw and persisted to 20 pcw. Myofibroblast 2 (*CXCL14^+^*, *KCNK17^+^*, *CT45A3^+^*, and *THBD*^*HI*^) and myofibroblast 3 (*CXCL14^+^*, *KCNK17^+^*, *CT45A3^-^*, and *THBD^-^)* were predominantly identified at 22 pcw (Figures 4A and 4B). Throughout development, myofibroblasts (*CXCL14^+^*, *KCNK17^+^*) were visualized surrounding the developing stalk region of the epithelium, suggesting a close signaling relationship (Figures 4D, 4H, S7K, and S7L). Although not detected in significant numbers in the scRNA-seq data until 22 pcw, we see myofibroblast 2 (PDGFRA^+^, THBD^HI^, and *NOTUM^+^*) around the stalk epithelium from 15 pcw (Figures 4D, S7L, S7N, and S7O), the same position as myofibroblast 1. The appearance of myofibroblast 2 is coincident with the acquisition of AT2 markers by the late-tip cells and may be a more mature state of myofibroblast 1. Myofibroblast 2 was enriched in gene expression associated with cell contractility and focal adhesions, as well as WNT signaling (Figures S7N and S7O). Co-expression of the Wnt-responsive genes *LEF1, NOTUM*, and *NKD1* suggests that myofibroblast 2 is responding to local Wnt expression (*WNT2* is high in alveolar fibroblasts) and producing the secreted Wnt inhibitor NOTUM, potentially to regulate local cell patterning. By contrast, myofibroblast 3 has higher expression of genes associated with ECM organization and a variety of signaling molecules, including *C7, RSPO2*, and *BMPER* (Figure S7N). Myofibroblast 3 was localized to the developing air sacs (Figure S7M), rather than the stalk epithelium, and is likely to be a precursor of the alveolar myofibroblasts.^17,39^

### Signaling niches in lung development

**Figure 5:**
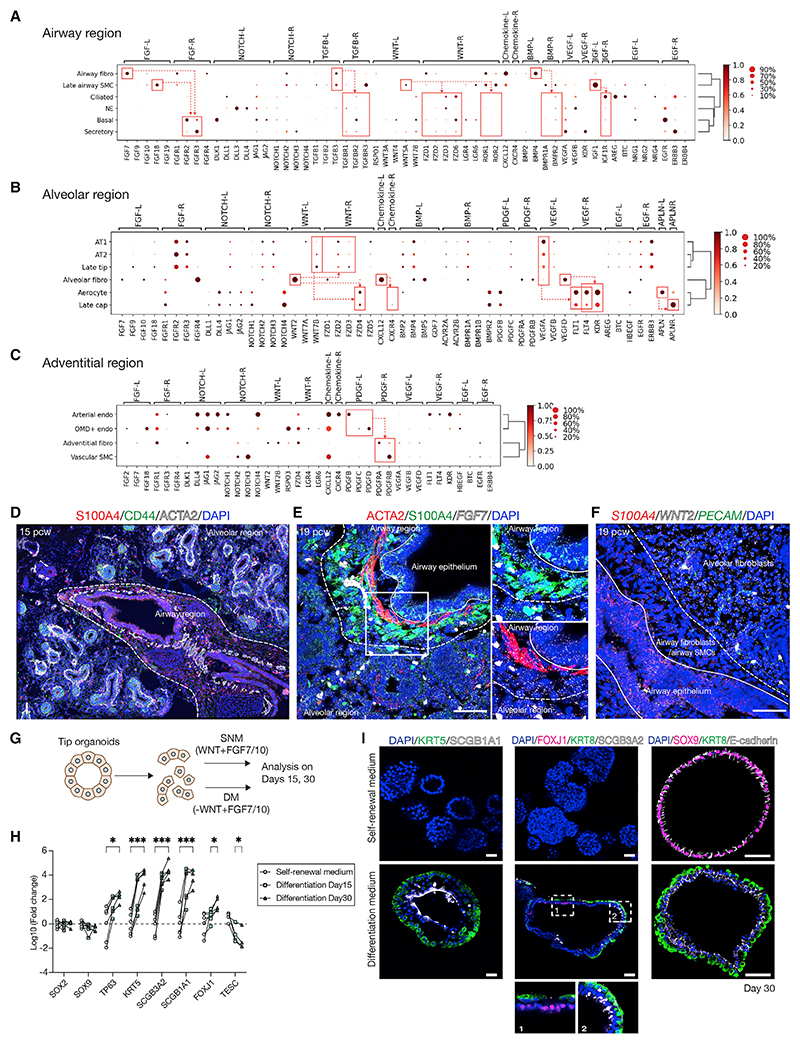
Signaling ligand-receptor interactions in specific niches (A–C) Curated ligand-receptor interaction predictions from CellPhoneDB in airway (A), alveolar (B), and adventitial (C) niches. Dot plots visualize gene expression by cell type; dashed arrows indicate the predicted direction of signaling from ligands to receptors. (D–F) Immunofluorescence/HCR. S100A4/*S100A4*, airway fibroblasts; ACTA2, ASM; CD44, tip epithelium; PECAM1, ECs. Airway fibroblasts/ASM form a boundary (dashed lines) between alveolar and airway regions. Lines are between airway fibroblasts/SMCs and airway epithelium. DAPI, nuclei. Scale bars, 50 μm. (G) Organoids were cultured in FGF7/10-containing medium, in the presence (self-renewal medium; SNM) or absence (differentiation medium; DM) of CHIR99021, for 30 days. (H) qRT-PCR quantification normalized to organoids cultured in SNM. Significance evaluated by two-way ANOVA with Tukey multiple comparison post-test; *p < 0.05, **p < 0.01, ***p < 0.001; n = 6 organoid lines. (I) Whole-mount immunofluorescence of lung organoids cultured in self-renewal medium (upper) and differentiation medium (lower). DAPI, nuclei. Scale bar, 25 μm.

We used CellPhoneDB^40^ to predict signaling interactions controlling cell fate. We focused on 15–22 pcw cells and, based on the localization of the major fibroblast populations (Figures 4E–4G), analyzed signaling within three niches.The airway niche includes airway fibroblasts, late airway SMCs, and airway epithelial cells. The alveolar niche includes alveolar fibroblasts, aerocytes, late Cap cells, late-tip cells, AT1, and AT2. Finally, the adventitial niche includes adventitial fibroblasts, arterial endothelium, OMD^+^ endothelium, and vascular smooth muscle cells. CellPhoneDB predicts numerous signaling interactions (Table S2) that we curated by plotting the expression of ligand-receptor pairs representing major signaling pathways (Figures 5A–5C). We observed expected interactions, including high levels of Notch ligands and receptors and CXCL12-CXCR4 signaling in the adventitial niche (Figure 5C).^41^ Similarly, expected signaling predicted in the alveolar niche included aerocytes to late cap cells (ALPN-ALPNR) and alveolar epithelial cells to microvascular ECs (VEGFA-FLT1/FLT4/KDR) (Figure 5B).^30,31^

Airway fibroblasts were predicted to signal via TGFβ3 and BMP4 to the airway epithelium, consistent with roles for these signals in human basal cell specification and differentiation.^42,43^ Airway fibroblasts and ASM were also predicted to signal to the epithelium via FGF7/18 to FGFR2/3 and non-canonical WNT5A to FZD/ROR (Figure 5A). By contrast, although FGF and WNT signaling interactions were also predicted in the alveolar niche, interactions were based on lower levels of *FGF* but higher levels of canonical *WNT2* and its receptor (Figure 5B). The predicted FGF and WNT signaling interactions in the alveolar niche and late-tip cells are consistent with the requirement of these factors for long-term self-renewal of human distal tip organoids.^8,29^ Tissue staining showed that although *FGF7* is expressed fairly ubiquitously, the airway fibroblasts and ASM form a distinct barrier between the airway epithelium and the *WNT2* expression (Figures 5D–5F). Based on these data, we predicted that removing canonical WNT but retaining FGF signaling would promote airway differentiation in the human distal tip organoids (Figure 5G). Indeed, we observed robust basal, secretory, and ciliated cell differentiation in response to FGF-containing medium (Figures 5H and 5I).

### scATAC-seq analysis identifies putative cell fate regulators

**Figure 6:**
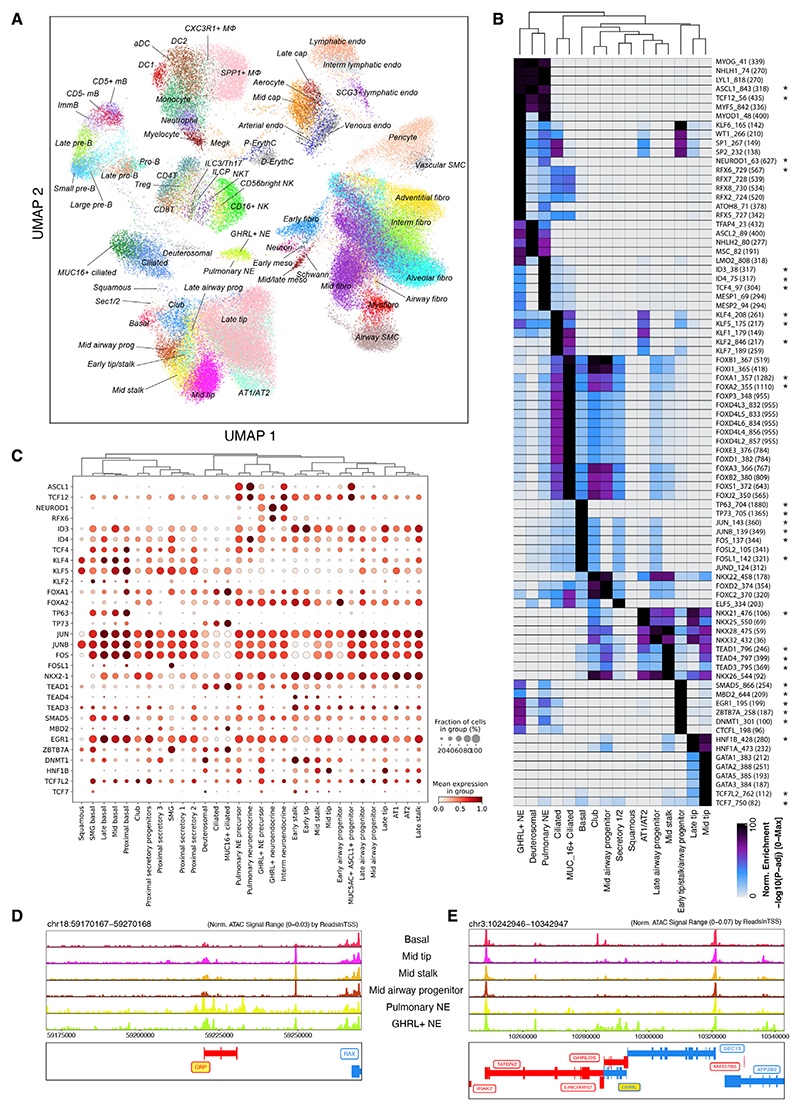
DNA accessibility and motif enrichment revealed by scATAC-seq (A) Single-cell DNA accessibility profiles mapped onto 2D UMAP. Colored for cell states. (B) Top 10 enriched motifs in the marker peaks among epithelial cell types/states. Statistical significance is visualized as a heatmap according to the color bar below. Transcription factors concordantly expressed based on scRNA-seq data are marked with asterisks. (C) Expression dot plot of the concordant transcription factors from (B) in epithelial cell types. (D and E) Read coverage tracks of *in silico* aggregated “pseudo-bulk” epithelial clusters over the GRP locus (D) and GHRL locus (E). See also Table S4.

Single cell ATAC-seq provides an independent method of assessing cellular-level gene regulation based on open chromatin regions and allows cell-type-specific TFs to be predicted. After tissue dissociation, the single-cell suspensions were split, and half of the cells were processed for nuclear isolation and scATAC-seq (Figure 1A). Following quality control and doublet removal, 67 scA-TAC-seq clusters comprising ~100K cells were obtained, and label transfer was used to annotate scATAC-seq clusters based on our scRNA-seq data (Figure 6A). Not every cell state detected by scRNA-seq was distinguishable by scATAC-seq, consistent with previous work.^13,44^ For example, separate early-tip, stalk, and airway progenitor clusters were discerned by scRNA-seq (Figure 2A), but a combined cluster with strong similarity to all three cell types was detected by scATAC-seq (Figure 6A). Nevertheless, there was broad agreement between the scRNA-seq and ATAC-seq data in terms of capturing cell types, including many of the novel/lesser-known cell types we identified by scRNA-seq (mid and late tip, mid and late airway progenitors, GHRL^+^ NE, MUC16^+^ ciliated, dueterosomal, airway fibroblasts, aerocytes, and SCG3^+^ lymphatic endothelial cells).

We analyzed TF binding motifs in the unique/enriched open chromatin regions in each cluster and plotted the top TF motifs per cell type (Table S4). As expected, TFs belonging to the same family are frequently enriched in the same cell type due to similarities in their binding motifs. This analysis revealed some expected TF signatures, for example TCF21 in the fibroblasts,^45^ GRHL, and FOXA1/2 in epithelium,^46,47^ and SOX17 in arterial endothelium.^48^ Examining epithelial cells and focusing on TFs expressed in the corresponding cell type in the scRNA-seq data (Figures 6B and 6C, marked by asterisk in 6B), TEAD motifs were enriched in mid-stalk cells, consistent with a key role for Yap,^49^ NKX2.1 in AT1/AT2 cells,^50^ KLF factors in secretory cells and AT1/AT2,^51^ and TP63 in basal cells.^52^ Unexpected TF signatures included HNF1B in late-tip cells and ZBTB7A in early-tip/stalk/airway progenitors. We focused on the pulmonary and GHRL^+^ NE cells, which cluster closely (Figures 2A and 6A). ASCL1 is required for mouse NE cell differentiation,^53,54^ and this motif is strongly associated with both pulmonary and GHRL^+^ NE cells (Figure 6B). However, both cell types also respectively have specific TF motifs including NEUROD1 and RFX6 in the GHRL^+^ NEs, and TCF4 and ID in the pulmonary NEs (Figure 6B). Consistent with this, there are distinct, unique regions of open chromatin, especially in the neighborhood of cell-type-specific genes such as GRP and GHRL (Figure 6D and 6E).

We have produced a high-resolution scATAC-seq dataset for the developing human lungs, which is highly consistent with our scRNA-seq data. Mining these data provides hypotheses for lineage-determining TFs in lung development.

### Transcriptional control of neuroendocrine cell subtype formation

**Figure 7:**
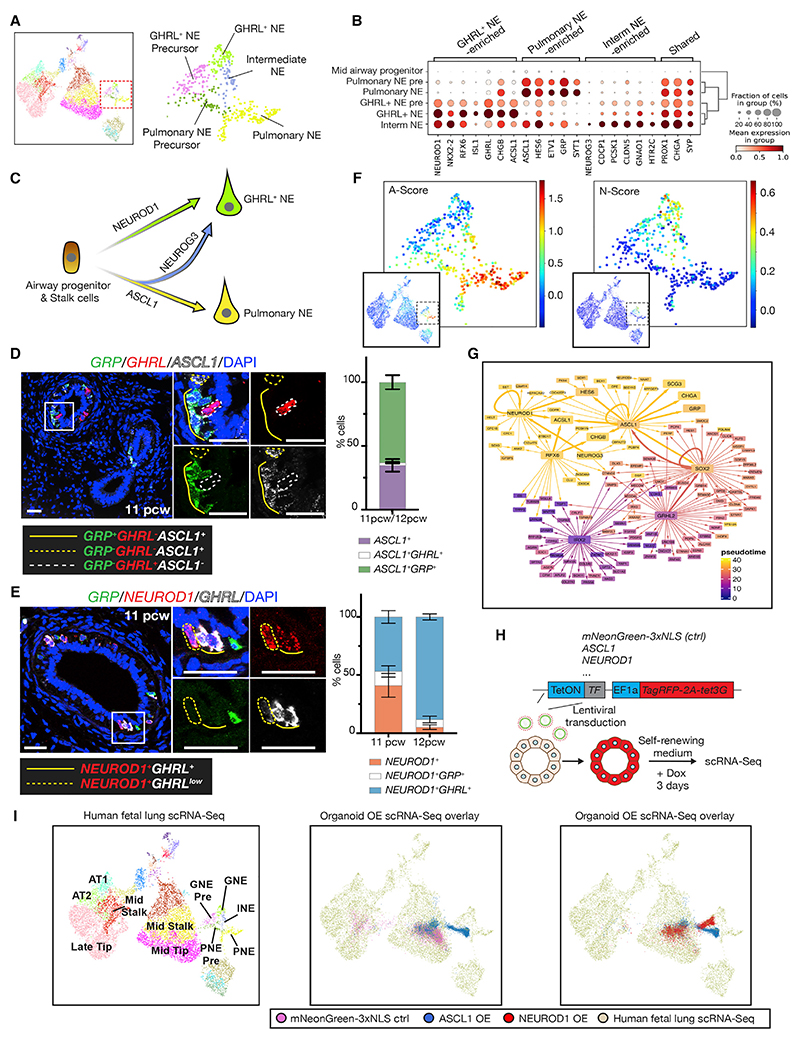
*ASCL1* and *NEUROD1* regulate the formation of two subtypes of neuroendocrine cells (A) Zoom-in UMAP plot of NE lineages. (B) Dot plot showing selected gene expression in NE lineages. (C) Schematic model of NE lineage formation. (D) Left: HCR, *GRP* (green), *GHRL* (red), *ASCL1* (white). Right: mean ± SEM of *ASCL1*^+^ cell types, N = 3 human fetal lungs, n = 243 *ASCL1*^+^ cells. (E) Left: HCR, *GRP* (green), *NEUROD1* (red), *GHRL* (white). Right: mean ± SEM of *NEUROD1*^+^ cell types: N = 2, 11 pcw human fetal lungs, n = 129; N = 3, 12 pcw human fetal lungs, n = 132. Scale bars, 25 μm. (F) Gene signature scoring of A-type and N-type SCLC features in the epithelial UMAP. (G) Scenic analysis of predicted TF network governing mid tip progenitor cells to pulmonary NE and GHRL+ NE. Trajectory and color coding match Figures S8A and S8B. (H) Organoids from 8 pcw human fetal lungs were transduced with Doxycycline (Dox)-inducible TF, or mNeonGreen-NLS, lentivirus. Transduced organoids were isolated by flow cytometry based on TagRFP expression, seeded in Matrigel for 10–13 days prior to Dox treatment. Organoid cells were harvested 3 days post-Dox for scRNA-Seq. N = 3 organoid lines. (I) Left: reference UMAP of primary human fetal lung epithelium. Mid and right: scRNA-Seq of organoids overexpressing *mNeonGreen-NLS, ASCL1*, or *NEUROD1* projected onto the primary data. See also Figure S8.

Pulmonary NE and GHRL^+^ NE cells share the expression of many TFs and open chromatin regions but are transcriptionally distinct. In our scRNA-seq data, they were both observed along a maturation trajectory and shared classical NE markers (*CHGA, SYP*), but differed in TF and hormone expression (Figures 7A and 7B). A third NE population (intermediate NE) clustered between pulmonary and GHRL^+^ NE cells with intermediate gene expression (Figures 7A and 7B), although it did contain a small number of cells expressing the unique marker *NEUROG3*. Pseudotime trajectory analysis suggested that pulmonary NE and GHRL^*+*^ NE cells were derived from airway progenitors/stalk cells and that intermediate NEs are an additional transition population (Figures S8A and S8B). Transition states between pulmonary NE and GHRL^+^ NE were observed in sections (Figure S8C). We therefore postulated that pulmonary NE precursors could acquire *NEUROG3* and convert to GHRL^+^ NE fate (Figure 7C), or vice-versa—GHRL^+^ precursors converting to pulmonary NE fate. In sections, *ASCL1* was co-expressed with *GRP*, but rarely with *GHRL*. We also observed *ASCL1* single-positive cells, likely representing pulmonary NE precursors (Figure 7D). *NEUROD1* was co-expressed with *GHRL* but also observed with *GRP* (Figure 7E), whereas *NEUROG3* was co-expressed with *ASCL1* and/or *NEUROD1*, supporting a role in a transition population (Figure S8D).

Differential expression of *ASCL1* and *NEUROD1* defines A- and N-type human SCLC, which likely derives from NE cells.^55^ Interestingly, these two TFs coincide with the scRNA-seq marker genes and scATAC-seq TF motif enrichment of our fetal NE cells (Figures 6B and 7B). We generated SCLC feature gene lists^18^ and performed gene signature scoring, showing that the A-type signature resembles pulmonary NEs, whereas the N type resembles GHRL^+^ NEs (Figure 7F). These data suggest that either there are two different NE cells of origin for human SCLCs or that SCLCs reuse developmental mechanisms, as suggested by some mouse models.^56^ We have been unable to detect GHRL^+^ NEs in the adult airways using HCR (5 biological replicates). However, a small number of GHRL^+^ cells are present within a tuft cell cluster in an integrated adult lung cell atlas containing 2.2 million cells,^57^ suggesting that GHRL^+^ NEs could be a rare cell state in the adult airways. Given their relevance to human disease states, we used our single-cell atlas to predict NE lineage-defining TFs and test these using our organoid system. We reasoned that overexpression of lineage-defining TFs in lung tip organoids^8,58^ would promote cell-type-specific differentiation.

Multiple TFs were differentially expressed between pulmonary NE and *GHRL^+^* NE cells (Figure 7B). We used SCENIC analysis of gene regulatory networks (GRNs)^59^ along a predicted airway progenitor to GHRL^+^ NE trajectory (Figures S8A and S8B) to identify putative lineage-defining TFs (Figure 7G). ASCL1, NEUROD1, and NEUROG3 all emerged as potential key nodes cell differentiation in various organs.^53,54,60,61^ We also selected the *GHRL^+^* NE-specific *RFX6* (Figure S8E) and *NKX2.2* (Figure 7B), the pan-NE *PROX1* (Figure 7B), and, as controls, the basal cell-specific TFs *DeltaNTP63, TFAP2A, PAX9*, and *mNeonGreen-3xNLS*. Overexpression of *PROX1* or *NKX2-2* did not result in NE gene upregulation based on qRT-PCR (data not shown), and these TFs were not followed up. The other factors resulted in increased expression of basal or NE markers compared to *mNeonGreen-3xNLS* controls, and the experiments were repeated using scRNA-seq. Individual TFs were overexpressed from a doxycycline-inducible construct for 3 days, and organoids were maintained in the self-renewing (tip cell-promoting) medium throughout to rigorously assay the lineage-determining competence of the TF (Figures 7H and S8F), followed by scRNA-seq.

When mapped to epithelial cells of our fetal lung atlas, the majority of the *mNeonGreen-3xNLS* expressing organoid cells projected to mid-tip or stalk cells as expected (Figure 7I), whereas overexpression of *DeltaNTP63* resulted in basal cell-like lineages (Figure S8G) consistent with a previous report.^62^ Overexpression of *RFX6, TFAP2A*, or *PAX9* did not result in the predicted lineage progression at a transcriptome level (Figure S8G). However, *ASCL1*-overexpressing organoids progressed into pulmonary NE precursors (Figure 7I), and *NEUROD1* overexpression promoted differentiation into *GHRL*^+^ NE precursors (Figure 7I). *NEUROG3* overexpression also led to *GHRL*^+^ NE precursor formation (Figure S8G), suggesting that the *GHRL^+^* NE lineage is the destination of the intermediate NE population (Figure 7C).

The 5′ differences between the transgenes and endogenous TFs allowed us to distinguish these transcripts and infer gene regulation hierarchy. We observed autoregulation of *ASCL1, NEUROD1, NEUROG3*, and *RFX6* (Figure S8H). By contrast, *NKX2-2* and *PROX1* were upregulated by other TFs, indicating they are relatively low in the hierarchy (Figure S8H). *NKX2-2* and *PROX1* expression in the organoid assay matched their expression in NE cells *in vivo* (Figures 7B and S8H), showing that this assay recapitulated key features of the TF network. These experiments tested GRN predictions from the single-cell atlas, confirmed the predicted lineage trajectory, and provided a foundation for studying human SCLC. This is significant given that there is no evidence that GHRL^+^ NE cells are present in mice,^18^ making the use of mouse models difficult.

## Discussion

Using a combination of single-cell and spatial approaches, we have identified 144 cell types, or states, in the developing human lungs across the 5–22 pcw period. We take advantage of a known proximal-distal gradient in epithelial differentiation to identify progenitor and differentiating states in the developing airway, including a neuroendocrine cell subtype related to SCLC. Moreover, analysis of the mesenchymal compartment identified three niche regions with distinct signaling interactions, allowing us to identify signaling conditions that are sufficient for airway differentiation of human embryonic lung organoids. We tested GRN predictions for NE cell differentiation in an organoid system, allowing us to identify lineage-defining TFs and provide directionality to the inferred differentiation trajectory. This study provides a paradigm for combining single-cell datasets with spatial analysis of the tissue and functional analyses in a human organoid system to provide mechanistic insights into human development.

Our data suggest that at all stages of lung development, cells exit the tip and enter a stalk state prior to differentiation. We propose that human alveolar epithelial differentiation also follows this model, using a tip-stalk-AT2 or AT1 fate decision pattern (Figure 3). This is different to the prevailing cellular models of mouse alveolar development: early cell fate restriction^17,63^ and bipotent progenitors with AT1/2 characteristics.^64^

Airway, adventitial, and alveolar fibroblasts are localized in distinct niche regions and participate in different signaling interactions. Airway and adventitial fibroblasts both express unique combinations of signaling molecules and also form physical barriers between the neighboring airway epithelium or vascular endothelium and the widespread alveolar fibroblasts (Figures 4 and 5). Similarly, we characterize a population of myofibroblasts that contacts the developing epithelial stalk region and expresses high levels of the secreted Wnt-inhibitor, *NOTUM* (Figure S7O), whereas alveolar fibroblasts express high levels of the canonical *WNT2* ligand (Figure 4). In a separate study, using surface markers identified in this single-cell atlas, we specifically isolated alveolar fibroblasts and myofibroblast 2 cells for co-culture experiments with late-tip organoids.^29^ Those experiments confirmed that a three-way signaling interaction between alveolar fibroblasts, myofibroblast 2 cells, and late-tip cells can control human AT2 spatial patterning.

We find that GHRL^+^ NE cells are transcriptionally similar to the NEUROD1^+^ N subtype of SCLC (Figure 7). Our functional analyses of NE cell differentiation in organoids will provide tools to test these hypotheses. Mouse studies show that fetal transcriptional and chromatin cell states are accessed during the normal process of tissue regeneration and may contribute to neoplasm in chronic inflammation.^65,66^ Detailed ATAC-seq datasets are not yet available for human lung disease. Our high-quality ATAC-seq atlas will provide a baseline for further analyses when adult chromatin accessibility lung atlases are published. In summary, our multi-component atlas is a community resource for future analyses of human development, regeneration, and disease.

### Limitations of the study

We provide a carefully annotated, descriptive cell atlas resource. Many conclusions are derived from trajectory inference or TF binding site analyses and require future validation. Trajectory inference analyses are largely based on transcriptomic similarities without ground-truth directionality, or are unable to handle complex expression kinetics in groups of genes.^67^ For these reasons, we fed Monocle3^68^ with starting and end points guided by known biological features of the data (age and spatial arrangement of cells). Furthermore, validation assays for lineage analysis in human systems rely on *in vitro* experiments. These usually define differentiation competence and do not necessarily mean that a specific differentiation route occurs *in vivo*. The clustering of our scRNA-seq and scATAC-seq data are in broad agreement. However, many motifs enriched in cell-type-specific peaks belong to TFs not detected by scRNA-seq. This discordance might be due to differing sensitivity of the two assays, transcription factor latency, and the incompleteness of the motif databases.

We have compared the identity of fetal and adult human lung cells and have seen many fetal-adult similarities. Nevertheless, there are approximately three decades between the oldest fetal and youngest adult human lung samples sequenced, including a rapid period of postnatal growth and morphogenesis, puberty, and unknown infections/environmental insults. It will be important to sequence additional lungs and, when possible, to fill the age gap. Moreover, our mouse-human fetal lung cell comparisons are affected by both technical (experimental protocols and annotation granularity) and biological differences (size and gestation rate). It will be informative in the future to make comparisons with a range of fetal lungs, including larger, long-developing species such as pig and sheep, to distinguish between differences due to species, size, and gestation period.

## Star+Methods

### Key Resources Table

**Table:** 

REAGENT or RESOURCE	SOURCE	IDENTIFIER
Antibodies		
Mouse monoclonal anti-ACTA2	Thermo Fisher Scientific	Cat#MA1-06110; RRID: AB_557419
PE-conjugated Mouse monoclonal anti-THBD (CD141)	BioLegend	Cat#344104; RRID: AB_2255842
Rabbit monoclonal anti-PDGFRA	Cell Signaling Technology	Cat#3174; RRID: AB_2162345
Rabbit polyclonal anti-S100A4	Proteintech	Cat#16105-1-AP; RRID: AB_11042591
APC-conjugated Rat monoclonal anti-CD44	Thermo Fisher Scientific	Cat#17-0441-82; RRID:AB_469390
Rabbit polyclonal anti-SOX9	Merck	Cat#AB5535; RRID:AB_2239761
Sheep polyclonal anti-PDPN	R&D systems	Cat#AF3670; RRID:AB_2162070
Rat monoclonal anti-E-cadherin	Thermo Fisher Scientific	Cat#13-1900; RRID:AB_2533005
Chicken polyclonal anti-KRT5	BioLegend	Cat#905901; RRID:AB_2565054
Rabbit polyclonal anti-SCGB1A1	Proteintech	Cat#10490-1-AP; RRID:AB_2183285
Mouse monoclonal anti-FOXJ1	Thermo Fisher Scientific	Cat#14-9965-80; RRID:AB_1548836
Rabbit monoclonal anti-SCGB3A2	Abcam	Cat#14-9965-80; RRID: N/A
Mouse polyclonal anti-SCGB3A1	Novus Biological	Cat#MAB27901; RRID:N/A
Biological samples		
Organoid line: HDBR-L 13393, 15909	HDBR London	N/A
Organoid line: BRC 1943, 1915, 2174, 2315, 2316	Brain Repair Center, University of Cambridge	N/A
Chemicals, peptides, and recombinant proteins		
Proteinase K solution	Thermo Fisher Scientific	Cat#AM2546
N2 supplement	Thermo Fisher Scientific	Cat#17502001
B27 supplement	Thermo Fisher Scientific	Cat#12587001
N-acetylcysteine	Merck	Cat#A9165
EGF	PeproTech	Cat#AF-100-15
FGF10	PeproTech	Cat#100-26
FGF7	PeproTech	Cat#100-19
Noggin	PeproTech	Cat#120-10C
R-spondin	Stem Cell Institute, University of Cambridge	
CHIR99021	Stem Cell Institute, University of Cambridge	
SB431542	bio-techne	1614
cAMP	Merck	B5386
IBMX	Merck	I5879
Y-27632	Merck	688000
Dexamethasone	Merck	D4902
Doxycycline	Merck	D9891
Critical commercial assays		
Chromium Single Cell V(D)J Kits (v1)	10X genomics	
Visium Spatial Gene Expression Slide & Reagents Kit	10X genomics	
Chromium Next GEM Single Cell ATAC Kits (v1)	10X genomics	
In-Fusion® HD Cloning Plus	Takara	638910
Deposited data		
scRNA-seq and scV(D)J of lung tissue	ArrayExpress	E-MTAB-11278
scRNA-seq of lung organoids	ArrayExpress	E-MTAB-11267
Visium spatial transcriptomics	ArrayExpress	E-MTAB-11265
scATAC-seq of lung tissue	ArrayExpress	E-MTAB-11266
Oligonucleotides		
Primer: TCR *γ*/*δ* library PCR1-R1_hTRDC: AGCTTGACAGCATTGTACTTCC	Mimitou et al.^70^	N/A
Primer: TCR *γ*/*δ* library PCR1-R1_hTRGC TGTGTCGTTAGTCTTCATGGTGTTCC	Mimitou et al.^70^	N/A
Primer: TCR *γ*/*δ* library PCR2-R2_hTRDC TCCTTCACCAGACAAGCGAC	Mimitou et al.^70^	N/A
Primer: TCR *γ*/*δ* library PCR2-R2_hTRGC GATCCCAGAATCGTGTTGCTC	Mimitou et al.^70^	N/A
SI-PCR primer: AATGATACGGCGACCACCG AGATCTACACTCTTTCCCTACACGACGC*T*C	Mimitou et al.^70^	N/A
Recombinant DNA		
Plasmid: pLenti-tetON-KRAB-dCas9-DHFR- EF1a-TagRFP-2A-tet3G	Sun et al.^58^	Addgene: #167935
Plasmid: pLenti-tetON-mNeonGreen-3XNLS-EF1a- TagRFP-2A-tet3G	this manuscript	N/A
Plasmid: pLenti-tetON-ASCL1 -EF1a- TagRFP-2A-tet3G	this manuscript	N/A
Plasmid: pLenti-tetON-NEUROD1-EF1a- TagRFP-2A-tet3G	this manuscript	N/A
Plasmid: pLenti-tetON-NEUROG3-EF1a- TagRFP-2A-tet3G	this manuscript	N/A
Plasmid: pLenti-tetON-RFX6-EF1a- TagRFP-2A-tet3G	this manuscript	N/A
Plasmid: pLenti-tetON-TFAP2A-EF1a- TagRFP-2A-tet3G	this manuscript	N/A
Plasmid: pLenti-tetON-DeltaNP63- EF1a-TagRFP-2A-tet3G	this manuscript	N/A
Plasmid: pLenti-tetON-PAX9-EF1a- TagRFP-2A-tet3G	this manuscript	N/A
Plasmid: pLenti-tetON-NKX2-2- EF1a-TagRFP-2A-tet3G	this manuscript	N/A
Plasmid: pLenti-tetON-PROX1- EF1a-TagRFP-2A-tet3G	this manuscript	N/A
Software and algorithms		
python-genomics	this manuscript	https://github.com/brianpenghe/python-genomics
Seurat3-plus	this manuscript	https://github.com/brianpenghe/Seurat3-plus
ImageJ (version: 2.1.0)	101	https://imagej.nih.gov/ij/; RRID:SCR_003070
GraphPad Prism software (version: 9.1.0)	GraphPad Software Inc.	GraphPad Prism (https://graphpad.com); RRID:SCR_015807
FlowJo software (version: 10.0.0)	FlowJo, LLC	FlowJo (https://www.flowjo.com/); RRID:SCR_008520
Scanpy (version: 1.5.0, 1.8.1)	Wolf et al.^76^	https://github.com/theislab/scanpy
bbknn (version: 1.5.1)	Polanski et al.^84^	https://github.com/Teichlab/bbknn
Scvelo (version 0.2.3)	Bergen et al.^93^	https://github.com/theislab/scvelo
Monocle 3 (version: 1.0.0)	68,88	https://github.com/cole-trapnell-lab/monocle3
pySCENIC (version: 0.11.2)	59,94	https://github.com/aertslab/pySCENIC
ComplexHeatmap (version 2.6.2)	Gu et al.^90^	https://github.com/jokergoo/ComplexHeatmap
seriation (version: 1.3.0)	Hahsler et al.^91^	https://github.com/mhahsler/seriation
souporcell (version: 2.0)	Heaton et al.^102^	https://github.com/wheaton5/souporcell
ArchR (version: 1.0.1)	Granja et al.^99^	https://github.com/GreenleafLab/ArchR
cellxgene (version: 0.16.7)	Megill et al.^103^	https://github.com/chanzuckerberg/cellxgene
clusterProfiler (version: 3.18.1)	Yuetal.^100^	https://github.com/YuLab-SMU/clusterProfiler
STARsolo (version: 2.7.3a)	Kaminow et al.^72^	https://github.com/alexdobin/STAR/blob/master/docs/STARsolo.md
EmptyDrop	Lun et al.^73^	https://github.com/MarioniLab/DropletUtils
cellranger (versions: 3.0.2, 4.0.0)	10X genomics	https://github.com/10XGenomics/cellranger
cellranger-atac (version: 1.2.0)	10X genomics	https://github.com/10XGenomics/cellranger-atac
SoupX (version: 1.4.5)	Young etal.^104^	https://github.com/constantAmateur/SoupX
dandelion (version: 0.1.10)	Stephenson et al.^75^	https://github.com/zktuong/dandelion
Scrublet (version 0.2.1)	Wolock et al.^105^	https://github.com/swolock/scrublet
macs2 (version: 2.2.7.1)	Zhang et al.^106^	https://github.com/macs3-project/MACS
Space Ranger (version: 1.1.0)	10X genomics	https://support.10xgenomics.com/spatial-gene-expression/software/pipelines/latest/what-is-space-ranger
Seurat (version 3.2.2)	Stuart et al.^82^	https://github.com/satijalab/seurat
sklearn (version: 0.24.2)	Pedregosa et al.^98^	https://github.com/scikit-learn/scikit-learn
CellPhoneDB(version: 2.1.7)	Vento-Tormo et al.^80^	https://github.com/Teichlab/cellphonedb/

## Resource Availability

### Lead contact

Further information and requests for resources and reagents should be directed to and will be fulfilled by the lead contact, Emma L. Rawlins (elr21@cam.ac.uk).

### Materials availability

Human lung organoid lines used in this study are available from the lead contact, Emma L. Rawlins (elr21@cam.ac.uk), with a completed Materials Transfer Agreement.

### Data and code availability

- Sequencing data have been deposited at ArrayExpress and ENA and are publicly available. Accession numbers are listed in the key resources table. Processed sequencing data and microscopy data reported in this paper are available at https://fetal-lung.cellgeni.sanger.ac.uk/. ATAC-seq pseudobulk coverage profiles can be browsed at https://genome.ucsc.edu/s/brianpenghe/scATAC_fetal_lung20211206
- All original code has been deposited at GitHub and is publicly available as of the date of publication. Links are listed in the key resources table.
- Any additional information required to reanalyze the data reported in this work is available from the lead contact upon request.

## Experimental Model And Subject Details

### Human lung tissue

Human embryonic and fetal lung tissues were provided from terminations of pregnancy from Cambridge University Hospitals NHS Foundation Trust under permission from NHS Research Ethical Committee (96/085) and the MRC/Wellcome Trust Human Developmental Biology Resource (London and Newcastle, University College London (UCL) site REC reference: 18/LO/0822; Newcastle site REC reference: 18/NE/0290; Project 200419; www.hdbr.org). Sample age ranged from 4 to 23 weeks of gestation (post-conception weeks; pcw). Stages of the samples were determined according to their external physical appearance and measurements. Sample names and gestational ages are listed in Table S3. None of the samples used for the current study had any known genetic abnormalities. Sample gender was unknown at the time of collection, but molecularly-inferred sample gender is available on the web interface (https://lungcellatlas.org).

Ethical approval for the adult human lung samples was given by the South Central Hampshire B Research Ethics Committee (REC reference 18/SC/0514, IRAS project: 245471) administered through the University College London Hospitals NHS Foundation Trust. Human adult lung samples were also obtained from Royal Papworth Hospital Tissue Research Bank (REC reference: 18/EE/0269).

## Method Details

### Cell isolation for 10X single cell RNA and ATAC seq

Proximal and distal regions for human fetal lung samples ≥ 15 pcw were separated as indicated in Figure 1 and minced with scissors. Whole fetal lung samples <15pcw were directly minced with scissors. Minced tissues were transferred into a 15 mL Falcon tube and mixed with 5 mL of dissociation solution (collagenase, 0.125 mg/ml, Sigma, C9891-100MG; dispase, 1 U/ml, Merck, 4942078001; DNaseI, 0.1 mg/mL, Merck, D4527-10KU). The mixture was incubated in a shaker incubator at 37°C with horizontal shaking at 135 rpm for 30 min (after 15 min of incubation, the mixture was triturated with 10mL straight pipette). 5 mL of termination solution (2% fetal bovine serum in PBS) was added to terminate the digestion reaction. A brief spin at 100X g was performed to pellet large tissue pieces. The supernatant was passed through a 40 μm filter and cell samples extracted for the single cell RNA and ATACseq protocols. Any large undigested pieces were further trypsinized with 3 mL of 5X trypsin (Trypsin EDTA X10, Thermo Fisher Scientific, 15400054) for 3–6 min in 37°C water baths to further expose epithelial cells. The reaction was stopped using 5mL of termination solution, filtered through a 40 μm cell strainer and collected. Cells were pelleted at 500X g for 5 min at 4°C. If the pellets were red, a red blood cell (RBC) removal step was performed by resuspending cells in 1X RBC lysis buffer (Thermo Fisher, 00-4300-54) for 3 min at room temperature. RBC lysis buffer was neutralised with 10 mL of termination solution. The cell suspension was passed through a 40 μm filter again. For some of the trypsinized cells, a CD326 (EpCAM) MACS enrichment (Miltenyi Biotec, 130-061-101) was performed to further enrich epithelial cells. Cells were counted, pelleted and resuspended in appropriate volume with PBS/0.04%BSA and single cell RNA and ATAC seq was carried out using 10X Chromium Single Cell V(D)J Kits (v1) and Chromium Next GEM Single Cell ATAC Kits (v1), respectively.

### Human fetal lung organoid maintenance

Human fetal lung organoids were derived and maintained as previously described.^8^ In brief, human foetal lung tissues were treated with Dispase (8 U/ml Thermo Fisher Scientific, 17105041) at room temperature (RT) for 2 min to digest mesenchymal connections. Mesothelium and mesenchymal cells were carefully removed by needles. Branching epithelial tips were micro-dissected by needles, transferred into Matrigel (356231, Corning) and seeded in a 24 well low-attachment plate (M9312-100EA, Greiner) with 4–5 tips per 50 μL Matrigel dome per well. The plate was incubated at 37°C for 5–10 min until the Matrigel domes solidified. 600 μL of self-renewing medium containing: N2 (1: 100, Thermo Fisher Scientific, 17502001), B27 (1: 50, Thermo Fisher Scientific, 12587001), N-acetylcysteine (1.25 mM), EGF (50 ng/mL, PeproTech, AF-100-15), FGF10 (100 ng/mL, PeproTech, 100–26), FGF7 (100 ng/mL, PeproTech, 100-19), Noggin (100 ng/mL, PeproTech, 120-10C), R-spondin (5% v/v, Stem Cell Institute, University of Cambridge), CHIR99021 (3 μM, Stem Cell Institute, University of Cambridge) and SB 431542 (10 μM, bio-techne, 1614), was added. Organoids were cultured under standard tissue culture conditions (37°C, 5% CO2), maintained in self-renewing medium and passaged by mechanically breaking using P200 pipettes every 4–7 days.

### Human fetal lung organoid bronchiolar differentiation

The progenitor organoids were expanded in self-renewal medium in BME (Basement Membrane Extract, R&D Systems, 3533-010-02). For airway differentiation, the organoids were dissociated by TrypLE and cultured in the differentiation medium (AdvDMEM+++, 1X B27, 1X N2, 1.25 mM N-acetylcysteine, 100 ng/mL FGF10, 100 ng/mL FGF7, 50 nM Dexamethasone, 0.1 mM cAMP, 0.1 mM IBMX, 10 μM Y-27632) for 15–30 days.

### Isolation and airway differentiation of SCGB3A2+ distal and proximal airway cells

Human fetal lungs at 8–11 pcw were carefully separated, and tip/stalk, distal airway, and proximal airway regions were further dissected using fine forceps under a dissecting microscope (Figure S5G). The tissue fragments were enzymatically digested into single cells by treating them in dissociation solution containing 0.125 mg/mL Collagenase, 1 U/ml Dispase and 0.1 U/μl DNAase, in a rotating incubator for 20 min at 37°C. The cells were treated with 1X RBC lysis buffer (Thermo Fisher, 00-4300-54), and were enriched by CD326 MACS beads according to the manufacturer’s instructions. The enriched epithelial cells from distal and proximal regions were infected with a lentivirus habouring *SCGB3A2* promoter-driven EGFP with *EF1a* promoter driven-TagRFP. Next, the infected TagRFP cells were sorted by EGFP expression by FACS and analysed by qRT-PCR after 48 h (Figures S5G and S5H). The sorted distal and proximal SCGB3A2-GFP positive cells were cultured for 28 and 45 days in the airway differentiation medium.

### RNA extraction, cDNA synthesis, and qRT-PCR analysis

The cultured lung organoids were collected and lysed. Total RNA was extracted according to the RNeasy Mini Kit (Qiagen, 74004) procedure. cDNA synthesis was performed using High-Capacity cDNA Reverse Transcription Kit (Applied Biosystems, 4368814) and the synthesised cDNA was diluted 1:20 for the qRT-PCR reaction (SYBR Green PCR Master Mix; Applied Biosystems, 4309155). Primer sequence information is listed in Table S5. Data were presented as fold-change, calculated by ddCt method, using ACTB as a reference gene control.

### Human fetal lung organoid immunofluorescence

The differentiated organoids were released from the BME and fixed in 4% PFA at 4°C for 30 min. Then the organoids were washed in PBS, incubated in 0.3% PBTX (0.3% Triton X-100 in PBS) at 4°C for 1 h, and blocked (1% bovine serum albumin, 5% normal donkey serum, 0.3% Triton X-100 in PBS) at 4°C overnight. The organoids were incubated with primary antibodies: SCGB3A2 (1:800, Abcam, ab181853), KRT5 (1: 500, BioLegend, 905901), E-cadherin (1: 500; Thermo Fisher Scientific, 13-1900), SOX9 (1: 400; Merck, AB5535), SCGB1A1 (1: 800, Proteintech, 10490-1-AP), SCGB3A1 (1:200, Novus Biological, MAB27901), FOXJ1 (1: 300, eBioscience, 14-9965-80) at 4°C overnight. The organoids were washed by PBS and further incubated with secondary antibodies (donkey anti-chicken 488, 1: 1000, Jackson Immunoresearch, 703-545-155; donkey anti-mouse 594, 1: 1000, Invitrogen, A-21203; donkey anti-rabbit 594, 1: 1000, Invitrogen, A-21207; donkey anti-rat 647, 1: 1000, Jackson Immunoresearch, 712-605-153; donkey anti-rabbit 647, 1: 1000, Invitrogen, A-31573). After DAPI staining (1 μg/mL) at 4°C for 1 hour, the organoids were processed through a thiodiethanol series (25%, 50%, 75% and 97% v/v concentration in PBS) at 4°C for imaging.

### Plasmid cloning

cDNAs for genes ASCL1, NEUROD1, NEUROG3, RFX6 and PAX9 were purchased from Genscript. cDNAs for gene TFAP2A and mNeonGreen-3XNLS were gifts from Azim Surani’s Group. cDNA for DeltaNTP63 was purchased from IDT as a gBlock fragment. cDNA sequences were cloned into a Doxycycline inducible vector pLenti-tetON-KRAB-dCas9-DHFR-EF1a-TagRFP-2A-tet3G (Addgene: #167935)^58^ using XhoI and BamHI sites by Infusion cloning (Takara, 638910).

A promoter region (chr5:147,878,065 + 147,878,803; 739 bp) of *SCGB3A2* was amplified using primers: 5′-AATTGAATCCCA GGTTTTTCAAAAGACACT-3′ and 5′-GACAGTTATCTGGGATATTTTTCAGGAGTTT-3′. The amplicons were cloned into a lentiviral vector, pLenti-(promoter)-EGFP/EF1a-TagRFP by Infusion (Takara, 638909). Plasmids used in this study will be deposited to Addgene.

### Lentivirus packaging

We packaged the lentivirus as described previously.^58^ In brief, HEK293T cells were grown in 10-cm dishes to 70–80% confluence. Lentiviral vector (10 μg) was co-transfected with packaging MD2.G (3 μg, Addgene plasmid # 12259), psPAX2 (6 μg, Addgene plasmid # 12260) and pAdVAntage (3 μg, E1711, Promega) using Lipofectamine 2000 Transfection Reagent (11668019, Thermo Fisher Scientific) according to manufacturer’s protocol. Medium was refreshed the next morning. Lentivirus containing cell medium was harvested at 24 and 48 h after medium refreshing and pooled together. Cell fragments were removed by 300X g centrifugation. Supernatant was then passed through a 0.45 μm filter. Lentivirus was concentrated using Lenti-X™ Concentrator (631232, Takara) according to the manufacture’s instructions. Lentivirus pellets were dissolved in 400 μL PBS, aliquoted and frozen in −80°C.

### Lentivirus transduction

Lentivirus transduction was performed as previously described.^58^ In brief, human fetal lung organoids derived from 3 independent donors were incubated with prewarmed TrypLE for 10 min with trituration after 5 min. Organoid single cells and small fragments were collected, counted, pelleted and resuspended to around 100K cells/500 μL self-renewing medium with ROCKi (10 μM Y-27632). 0.5–2 μL of lentivirus was added and incubated overnight. Organoid cells were harvested the next morning, pelleted and re-seeded into Matrigel.

### Overexpression of transcription factors and scRNA-Seq

After 3 days of lentivirus transduction, organoids were dissociated by incubation with prewarmed TrypLE for 10 min with trituration after 5 min. TagRFP positive cells were sorted (20–40% of TagRFP positive rate), seeded back to Matrigel and allowed to recover for 10–12 days with self-renewing medium plus ROCKi (10 μM Y-27632). Organoids were treated with Doxycycline (2 μg/mL) for 3 days. Organoids were then fully dissociated into single cells by incubation with prewarmed TrypLE (Thermo Fisher Scientific, 12605028) for 15–20 min with trituration every 5 min. Organoid cells were counted, pelleted, resuspended in proper amounts of PBS/0.04%BSA and proceeded to scRNA-Seq according to 10X Chromium Single Cell V(D)J Kit manual.

### *In situ* hybridization chain reaction and immunofluorescence

*In situ* HCR v3.0 was performed according to the manufacturer’s protocol (Molecular Instruments.^69^ Probes were designed according to the manual, and amplifiers with buffers were supplied by Molecular Instruments. All the sequence information of the probes is listed in Table S5. In brief, the frozen human tissue sections fixed in 4% PFA/DEPC-treated PBS were cut into 20 μm slices and rinsed in nuclease-free ultrapure water, followed by 10 μg/mL proteinase K solution (Thermo Fisher Scientific, AM2546) for 2 min at 37°C. For *in situ* HCR with immunostaining, the tissue slices were permeabilized in 0.3% Triton-X/DEPC-treated PBS for 5 min at room temperature, avoiding the treatment of the proteinase K solution. Next, the tissue slices were incubated with 2 pmol of probes at 37°C overnight. After washing, the slices were treated with 6 pmol of the amplifiers at room temperature overnight. The amplifiers, consisting of a pair of hairpins conjugated to fluorophores, Alexa 488, 546, or 647, were used at final concentration of 0.03 μM. Then, excess hairpins were rinsed in 5X SSC (sodium chloride sodium citrate) solution containing 0.1% Triton X-100. Nuclei were counterstained with DAPI. For the immunostaining following the *in situ* HCR, the tissue slices were incubated with a blocking solution containing 5% NDS, 1% BSA, 0.1% Triton-X in DEPC-treated PBS at room temperature for 1 h after the hairpin amplification. After rinsing with DEPC-treated PBS, treated with primary antibodies against ACTA2 (1:500; Thermo Fisher Scientific, MA1-06110), THBD (1:100; PE-conjugated; BioLegend, 344104), PDGFRA (1:200; Cell Signaling Technology, 3174), S100A4 (1:200; Proteintech, 16105-1-AP), CD44 (1:200; Thermo Fisher Scientific, 17-0441-82), SOX9 (1: 200, Merck, AB5535), PDPN (1:200; R&D Systems, AF3670), or E-cadherin (1: 500; Thermo Fisher Scientific, 13-1900) overnight. Secondary antibodies were treated for 3 h at room temperature. The tissue was washed three times in DEPC-treated PBS at room temperature and counterstained with DAPI. Images were collected under Leica SP8 confocal microscope.

### Library generation and sequencing

Chromium Single Cell 5’ V(D)J Reagent Kits (V1.0 chemistry) were used for scRNAseq library construction. Gene expression libraries (GEX) and V(D)J libraries were prepared according to the manufacturer’s protocol (10X Genomics) using individual Chromium i7 Sample Indices. Libraries for gamma/delta TCR variable regions were amplified as previously described.^70,71^ GEX and V(D)J were pooled in 1:0.1 ratio respectively and sequenced on a NovaSeq 6000 S4 or Illumina HiSeq 4000 Flowcell (paired-end (PE), 150-bp reads) aiming for a minimum of 50,000 PE reads per cell for GEX libraries and 5,000 PE reads per cell for V(D)J libraries.

### Visium spatial transcriptomics

Fetal lung samples at 12–20 post conception week (pcw) from the HDBR, up to 0.5cm^3^ in size, were embedded in OCT and flashfrozen in dry-ice cooled isopentane. Twelve-micron cryosections were cut onto Visium slides, haematoxylin and eosin stained and imaged at 20X magnification on a Hamamatsu Nanozoomer 2.0 HT Brightfield. These were then further processed according to the 10X Genomics Visium protocol, using a permeabilization time of 18 min for 12–17 pcw samples and 24 min for 19 pcw and older samples. Images were exported as tiled tiffs for analysis. Dual-indexed libraries were prepared as in the 10X Genomics protocol, pooled at 2.25 nM and sequenced in 4 samples per Illumina Novaseq SP flow cell with read lengths of 28 bp for R1, 10 bp for i7 index, 10 bp for i5 index, 90 bp for R2.

### Reads mapping and quantification

scRNA-seq data were mapped with STARsolo 2.7.3a^72^ to the 10X distributed GRCh38 reference, version 3.0.0, derived from Ensembl 93. Cell calling was post-processed with an implementation of EmptyDrops^73^ extracted from Cell Ranger 3.0.2 (distributed as empty drops on PyPi). For transduced organoid cells, exogenous genes were added to the reference as appropriate for organoids, with the transgene sequence truncated (length(R2)-1) bp after the end of the synthetic promoter to avoid reads from endogenous transcripts being mapped onto transgenes. For single-cell V(D)J data, reads were mapped with Cell Ranger 4.0.0 to the 10X distributed VDJ reference, version 4.0.0. Visium reads were mapped with Space Ranger 1.1.0 to the 10X distributed GRCh38 reference, version 3.0.0, derived from Ensembl 93 for consistency with the single cell data. scATAC reads were mapped with Cellrangeratac 1.2.0 to reference GRCh38-1.2.0.

### VDJ analysis

Both TCR and BCR contigs contained in respective all_contigs.fasta and all_contig_annotations.csv files were re-annotated with igblastn (v1.17.1) using reference sequences curated from IMGT database (downloaded 01-Aug-2021) as per described with changeo (v1.0.0). For BCR contigs, heavy chain constant region calls were re-annotated using blastn (v2.12.0+) against curated sequences of CH1 regions corresponding to respective isotype classes from IMGT. BCR heavy chain V-gene alleles were corrected for individual genotypes using tigger (v1.0.0).^74^ Contigs were then filtered for basic quality control as described previously.^75^ Briefly, the following occurrences would lead to removal of contigs from further analysis: i) contigs were annotated with V, D, J or constant gene calls that are not from the same locus; ii) multiple long/heavy chain contigs present in the same cell; iii) there were only short/light chain contigs in a cell; and/or iv) there are multiple short/light chain contigs in a cell. Cells with multiple contigs were nevertheless retained if a) contigs were assessed to have identical V(D)J sequences but were assigned to a different contig by cellranger-vdj (pre-sumably due to differences in non-V(D)J elements); b) UMI count differences were large in which case the contig with the highest UMI count is retained; and c) only IgM and IgD were both assigned to a cell. These checks were all performed using dandelion^75^ singularity container (v0.1.10).

### Single-cell RNA-seq processing and cell type annotation

Count matrices were loaded into Scanpy and concatenated. Cells expressing no more than 200 genes, and genes detected in no more than 5 cells, were removed. Cells having more than 20% of their reads mapped to mitochondria were also discarded. Counts were then divided by total counts and multiplied by a factor of 10000, followed by log transformation, all implemented in Scanpy’s default setting^76^. $Y_{ij}=\ln\left(\frac{x_{ij}}{\Sigma_{i=1}^{n}x_{ij}}\cdot 10000+1\right)$, where *X*_*ij*_ is the raw count of i^th^ gene in j^th^ cell.

Feature genes were selected in three steps: For each sample, highly variable genes were calculated using Scanpy’s default settings that extract genes with highest dispersion (variance divided by mean) values of log-transformed counts. Next, highly correlated genes for each sample were extracted using the DeepTree algorithm described in,^12^ reimplemented in our python-genomics toolkit. Genes extracted in at least two samples were merged as the final feature gene list. The log-transformed counts of these genes were then scaled after cell-cycle scores were regressed out using Scanpy’s default scoring and regression functions. Using the top 50 PCs and 10 neighbors with resolution at 0.01, initial clustering was generated, yielding 10 major clusters (Figure S1C) corresponding to different compartments. These clusters were subsequently and recursively subclustered, curated and annotated manually (Figure S1D). Annotation was based on markers summarised in Table S1.

### Artefact evaluation and removal for scRNA-seq data

Doublets were evaluated using Scrublet in a batch-by-batch fashion (Figure S1G). To capture rare doublet clusters, we developed a method for Doublet Cluster Labeling (DouCLing, Figure S1E). Briefly, we calculated relative marker genes for each subcluster compared to other subclusters in the same parental large cluster. Then these marker genes were used to score all the cells in the atlas. If the top-scoring cells (above the mean score of the current subcluster) are mostly (>60%) from another large cluster, the clusters are flagged as doublet-like (Figure S1G’). We then removed doublet-like clusters based on these two methods with manual curation (Figure S1G”).

Maternally derived cells were evaluated based on SNP variations between the transcribed paternal genome in the fetuses and the maternal counterparts in the maternal cells. To do this, we indexed and pooled samples from the same donor into “Supersamples”. Then we applied Souporcell to compare known common variants captured in scRNA-seq reads, setting the sample number to 2. Supersamples without maternal cells would split into two equal-sized groups while other supersamples would putatively capture maternal cells as a minor genotype group (Figure S1F). Based on this analysis, maternal-like cells do not contribute to scRNA-seq clusters (Figure S1J) and were thus kept for downstream analysis. For libraries with two multiplexed donors, we only used the Souporcell workflow to demultiplex the donors without maternal genetic detection.

Low-quality cells would usually have a relatively high percentage of mitochondria reads (Figure S1I’) or a low number of genes detected (Figure S1I). Based on these we manually curated and removed low-quality clusters (Figure S1I”).

An additional four clusters of contaminants coming from other organs were further removed (Figure S1H). These were cardiomyocytes (ACTN2^+^ MYH6^+^),^77^ esophagus epithelial cells (SOX2^+^ TP63^+^ TRH^+^,^78^ APOA1^+^ APOA2^+^)^79^ and cytotrophoblasts from the placenta (PAGE4^+^ GSTA3^+^).^80^

### Visium spatial transcriptomics data analysis

Two methods were used side by side to predict cell compositions of the Visium datasets. Mapped Visium and filtered scRNA-seq data (removing cell types that have fewer than 20 cells) were both fed into the default pipeline of the cell2location algorithm,^81^ with the default detection alpha set to 20. The q05_cell_abundance was used as a conservative estimate of cell abundance in each voxel. This method was used to generate figure panels in this manuscript. In the alternative method, mapped Visium count matrices and scRNA-seq count matrices (after artefact removal) were both imported into Seurat 3^82^ and transformed using SCTransform,^83^ with mitochondria percentage of scRNA-seq data regressed out. Next, the scRNA-seq data were subsetted into a “pcw11,15,18” subgroup and a “pcw18,20,22” subgroup for cell-type prediction. The prediction was done for each Visium library using its corresponding scRNA-seq subgroup following the default label transfer pipeline of Seurat using the top 50 PCs. We provide the results of both methods on our data portal.

### Differential gene expression along trajectories

The single cell transcriptomics data was preprocessed using Scanpy^76^ version 1.8.1. The cell cycle effect was regressed out using scanpy.pp.regress_out and batch correction was performed using bbknn,^84^ before denoising the knn-graph using diffusion maps^85^ with scanpy.tl.diffmap and applying PAGA^86^ with scanpy.tl.paga to examine the connectivities between cell types. The final UMAPs were computed using the results of PAGA on Leiden^87^ clusters as previously described.^86^ Data and UMAPs were exported into R, and monocle3^68,88^ was used to find a principal graph and define pseudotime. Differentially expressed genes were then computed along pseudotime using a graph-based test (morans’ I)^88,89^ and the principal graph in monocle3, which allows identification of genes upregulated at any point in pseudotime. The results were visualised with heatmaps using the complexHeatmap^90^ and seriation^91^ packages, after smoothing gene expression with smoothing splines in R (smooth.spline, df = 12).

### CellPhoneDB analysis

Filtered single-cell RNA-seq data were partitioned into early- (5-6pcw), middle- (9–11) and late-stage (15–22) subsets and grouped into broad cell types. These datasets were used as input for CellPhoneDB^80^ (command: cellphonedb method statistical_analysis –database v2.0.0 –threads 20 –counts-data gene_name –project-name FetalLungBroad –subsampling –subsampling-log False –subsampling-num-cells = $TotalCellNumber –iterations = 10000 –result-precision = 4). Interaction pairs were manually curated from the outputs.

### Velocity analysis

Velocity analysis^92^ was performed using scvelo^93^ version 0.2.3. The preprocessed dataset was merged with spliced and unspliced read counts computed with velocyto, before using scvelo.pp.moments, scvelo.tl.velocity and scvelo.tl.velocity_graph to compute velocities using the stochastic mode in scvelo.

### Gene regulatory network analysis

The Scenic pipeline^59,94^ was used (pySCENIC version 0.11.2) to predict transcription factors and putative target genes regulated throughout neuroendocrine cell differentiation. First, gene regulatory interactions were calculated based on co-expression across the single cell dataset with GRNBoost2,^95^ followed by pruning interactions using known TF binding motifs and the construction of dataset specific regulatory modules (regulons).^96^ Regulons were then scored in each individual cell using AUCell. Cells of the neuroendocrine differentiation trajectory computed with monocle3 (as described above) were selected. The regulon target genes were filtered for differentially expressed genes along pseudotime for this trajectory. A network of TFs and target genes was then constructed by linking individual regulons.

### Comparing fetal neuroendocrine transcriptome with SCLC

A-type and N-type signatures were selected from previous data ‘ASCL1High and NEUROD1High Gene Signatures and the Stratified Primary Tumor Samples’.^18^ Top 10 genes with the highest fold enrichment were selected to score epithelial cells, using Scanpy’s tl.score_genes function.

### Comparing scRNA-seq datasets of the fetal lung and other studies

Annotated scRNA-seq adult lung datasets,^14^ the multi-organ scRNA-seq dataset,^13^ and the mouse scRNA-seq dataset^17^ were downloaded. Orthologs were translated from mouse to human counterparts using ENSEMBL biomart. scVI^97^ was used to integrate our fetal lung scRNA-seq and the Madissoon et al. and Zepp et al. data (human-mouse orthologs only), with sample IDs and project IDs both included as categorical covariate keys (other parameters: n_latent = 30, encode_covariates = True, dropout_rate = 0.2, n_layers = 2, early_stopping = True, train_size = 0.9, early_stopping_patience = 45, max_epochs = 400, batch_size = 1024, limit_ train_batches = 20, use_gpu = True). The latent variables calculated by xcVI were fed into Scanpy’s pl.correlation_matrix function to calculate and visualise correlation scores. A logistic regression model was trained based on the fine-grained cell-types for each of the multi-organ data, using sklearn.linear_model.LogisticRegression.^98^ The trained model was then used to predict the cell types of single-cell transcriptomic profiles of the fetal lung (Figure S1O).

### Single-cell ATAC-seq processing and annotation

Cellranger-atac outputs were loaded into and processed by ArchR.^99^ The top 50 dimensions were used for LSI and no batch effect was carried out to preserve weak biological features. Doublets were removed using ArchR’s default settings. Cells with TSSEnrichment score <8 or ReadsInTSS <1000 were discarded. Initial clustering was performed at resolution = 0.01 to be consistent with scRNA-seq, resulting in 7 large clusters corresponding to compartments. These clusters were further subclustered, similar to the workflow for scRNA-seq.

To annotate cell types and doublets, the annotated scRNA-seq dataset was loaded into Seurat3 by Seurat3-plus and integrated to scATAC-seq data using ArchR. The predicted cell type/state labels were used as a major reference for annotation. Clusters mapped to scRNA-seq doublet clusters were removed. Clusters with high fractions of blacklisted reads were also manually discarded.

Peaks were then called based on pseudo-bulk coverages by macs2. Marker peaks were calculated with default settings. Motifs from *cis*-bp database that are enriched in marker peaks were calculated and plotted.

### Comparing organoid scRNA-seq with fetal lung scRNA-seq

Organoid scRNA-seq data were imported and filtered in the same way as described above. Organoid data were then projected onto fetal tissue data by Scanpy’s tl.ingest function. Donors were demultiplexed using Souporcell with k = 3 donors, based on common variants.

## Quantification And Statistical Analysis

### HCR image analysis

*In situ* HCR images were analyzed using ImageJ (https://imagej.nih.gov/ij/) for quantification and statistical analysis. Cells expressing airway lineage markers along distal to proximal airway axis at different ages, mid (10–12 pcw) and late (15–21 pcw) stages, were counted (Figure S4B). For measuring the proportion of proximal secretory lineage cells within proximal cartilaginous airway regions, the fetal tissue sections at 10–12, 15–16, and 19–21 pcw were analysed based on expression patterns of *SCGB3A2, SCGB3A1*, and/ or *SCGB1A1* (Figure S4C). Mean, SD, 1-way ANOVA, and 2-way ANOVA were calculated using the Prism software (GraphPad Prism). Significance was evaluated by 1 or 2-way ANOVA with Tukey multiple comparison post-test; ns = not significant, *p < 0.05, **p < 0.01, ***p < 0.001, ****p < 0.0001.

### Statistical analysis for cell-type composition biases

Chi-squared test of independence was performed for sample gestation age, cell-cycle stage and proximal/distal dissection regions against cell type categories. For proximal/distal biases, Fisher exact test was used for each cell type and Benjamini-Hochberg correction was performed for multiple testing.

We also visualised the effect size for cell composition biases over developmental age and proximal/distal dissection in Figure S1O. After removing the clusters that are specific to the very early stages in low abundance (such as neuronal clusters), cell number counts were normalised against total number counts per stage. The mean developmental stage for each cluster was calculated based on the empirical distribution based on the aforementioned normalised counts, denoted by x. The weighted probability y of proximal representation was calculated as the frequency of cells from proximal samples normalised against total numbers of cells from proximal samples, ignoring whole-lung samples. The x and y values were calculated for Figure S1O.

$$\begin{array}{c} x_{t}=\sum_{s=5}^{22}\;sp_{s|t},\;where\;p_{s|t}=\frac{C_{t,s}/\sum_{t=t1}^{tn}C_{t,s}}{\sum_{s=5}^{22}\left(C_{t,s}/\sum_{t=t1}^{tn}C_{t,s}\right)}\\ y_{t}=\frac{C_{t,\;prox\;}/C_{prox\;}}{C_{t,\;prox\;}/C_{prox\;}+C_{t,\;dist\;}/C_{dist\;}} \end{array}$$

where (x_t_,y_t_) are the x and y coordinates of a cell type t, s is the post conception week, C_t,s_ is the number of cells labelled as cell type t at stage s, C_t,prox_ and C_t,dist_ are the numbers of cells labelled as cell type t coming from proximal and distal samples, respectively.

### Marker gene calculation

Ambrient RNA was removed with SoupX 1.4.5 with default parameters. Using the corrected count matrices, Scanpy.tl.rank_genes_groups was applied with default settings but keeping all the genes. These ranked genes were then filtered using Scanpy.tl.filter_rank_genes_groups with max_out_group_fraction = 0.25 and min_fold_change = 2. To compare specific cell types in the same compartment, Scanpy.tl.rank_genes_groups was applied for each cell type with only the other cell types of this compartment as a reference. Over-representation analysis (hypergeometric test) with gene sets from GO BP, KEGG and MSigDB was performed using the clusterProfiler R package.^100^

## Supplementary Material

Table S1

Table S2

Table S3

Table S4

Table S5

Supplemental Figures

## Highlights

- Spatiotemporal atlas of human lung development identifies 144 cell types/states
- Tracking the developmental origins of multiple cell types, including new progenitors
- Functional diversity of fibroblasts in distinct anatomical signaling niches
- Experimental validation of TFs controlling neuroendocrine cell heterogeneity

## In brief

Multiomic analysis of human fetal lungs from 5–22 post-conception weeks unveils cell-lineage trajectories across different cell types during development and will provide fresh insights into lung disease progression in adults.
