# Supplementary material for: A human fetal lung cell atlas uncovers proximal-distal gradients of differentiation and key regulators of epithelial fates
Source: Cell. Author manuscript; Available in PMC 2025 Dec 4. (PMC7618435; doi:10.1016/j.cell.2022.11.005)
Supplement: Supplemental Figures [file EMS211310-supplement-Supplemental_Figures.pdf]

CellPress
OPEN ACCESS

Supplemental figures

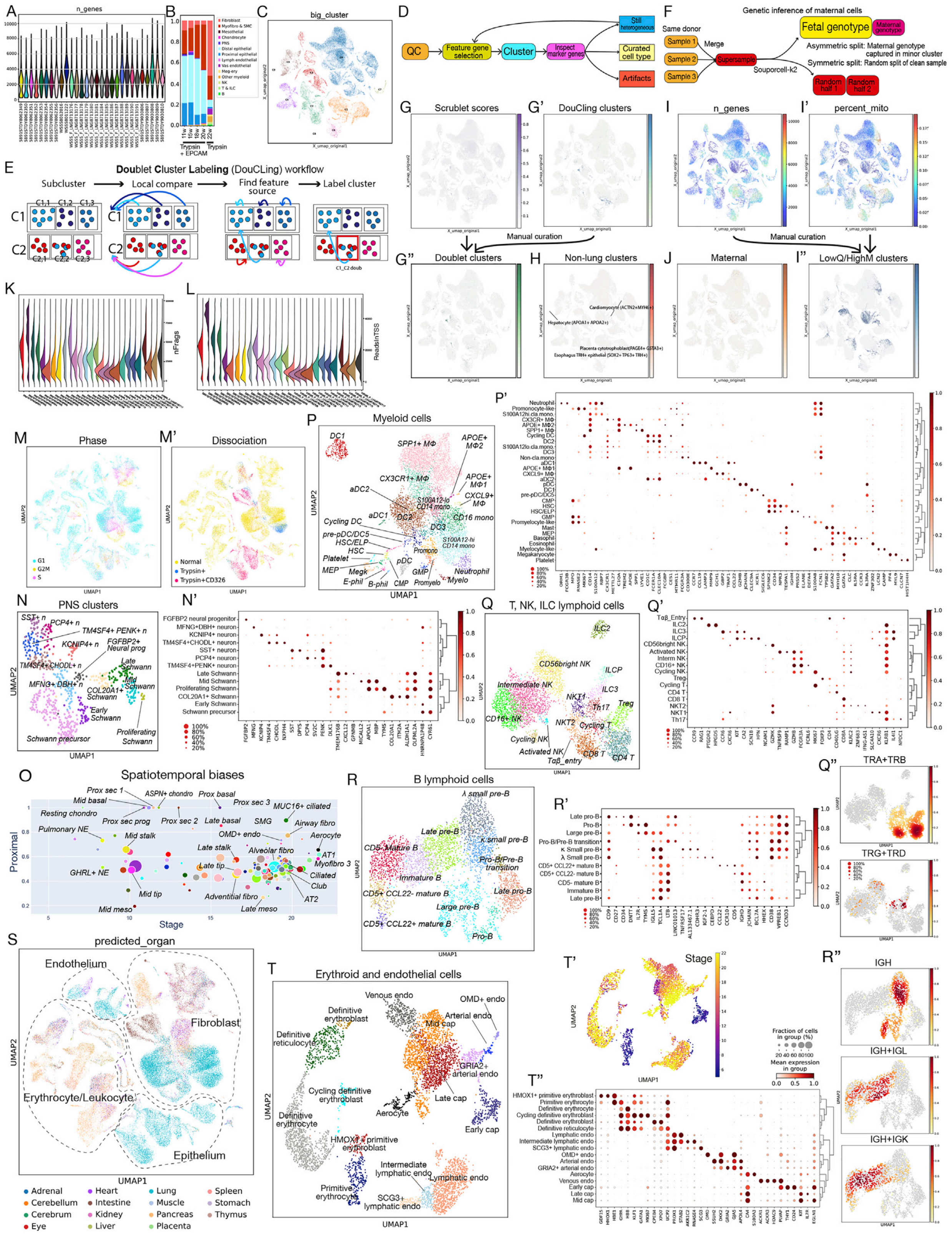

(legend on next page)

Figure S1. Quality control for scRNA-seq and scATAC-seq data and clustering overview of 144 cell types or cell states, related to Figures 1 and S7

(A) Distributions of the number of genes detected per cell, grouped by 10X libraries.
(B) Proportions of broad cell types in samples treated with Trypsin and Trypsin plus EPCAM enrichment following color codes in Figure 1C.
(C) Initial clusters of data-separating compartments, before subclustering.
(D–F) Workflows of the recursive subclustering method (D), the Doublet Cluster Labeling (DouCLing) method to identify doublet-driven clusters (E), and inference of maternal cells using Souporcell (F).
(G) Doublet scores by Scrublet (G), inferred doublet clusters by DouCLing (G'), and cells in curated doublet clusters (G'').
(H) Cells in clusters of cells coming from other organs. Marker genes in parentheses.
(I) Number of genes (I), percentage of mitochondrial reads (I'), and cells in curated low-quality cell clusters (I'') were projected on UMAP.
(J) Inferred maternal cells.
(K and L) scATAC-seq quality metrics of fragment detection per cell (K) and reads mapped in transcription-start sites (L).
(M) All of the curated 144 clusters of single cells projected on UMAP space of transcriptomes, colored by inferred cell-cycle phase (M), and dissociation/enrichment strategy (M').
(N) Cells from the initial PNS cluster (C7) projected on UMAP space of transcriptomes, colored by cell type/state (N) and selected feature genes of cell types/states (N') in the initial PNS cluster.
(O) Spatiotemporal biases of cell types. Cell types are shown as dots, with *x* representing the weighted average of developmental stages, *y* representing the score of proximal enrichment, and the size corresponding to the cluster size.
(P) UMAP embedding (P) and dot plots (P') of myeloid cell types/states.
(Q) UMAP embedding (Q), dot plots (Q'), and enrichment of each class of immune receptors based on abTCR, gdTCR, and BCR-enriched scRNA-seq (Q''), in T, NK, and ILC lymphoid cell compartments.
(R) UMAP embedding (R), dot plots (R'), and enrichment of immune receptors based on BCR-enriched scRNA-seq (R''), in B lymphoid cell compartment.
(S) Predicted organ-of-source with highest scores for cells shown in Figure 1, based on the reference atlas in Cao et al.[13]
(T) UMAP visualization of erythroid and endothelial cells colored by cell types/states (T), stages (T'), and dot plot describing differential marker gene expression level by cell type (T'').

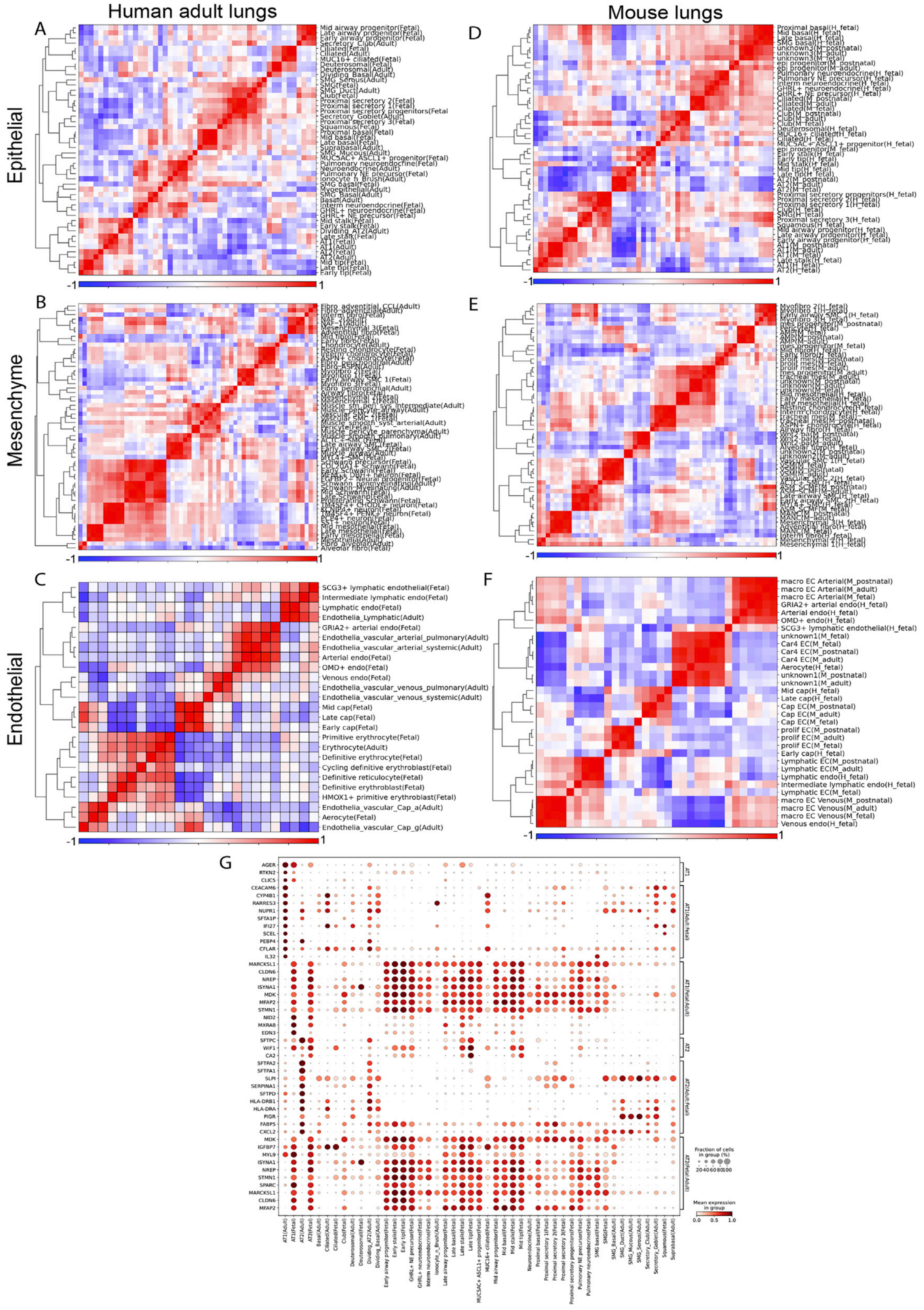

(legend on next page)

Figure S2. Comparing fetal lung scRNA-seq with adult human and mouse lung scRNA-seq, related to Figure 1

(A–F) Correlations of scVI latent variables between human fetal lung cell clusters and those of previously annotated adult cell clusters[1] (A–C) and mouse lung cell clusters[2] (D–F), focusing on epithelial (A, D), fibroblast (B, E), and endothelial (C, F) compartments.

(G) Expression of genes shared or unique to fetal/adult lung AT1/AT2 cell clusters.

CellPress
OPEN ACCESS

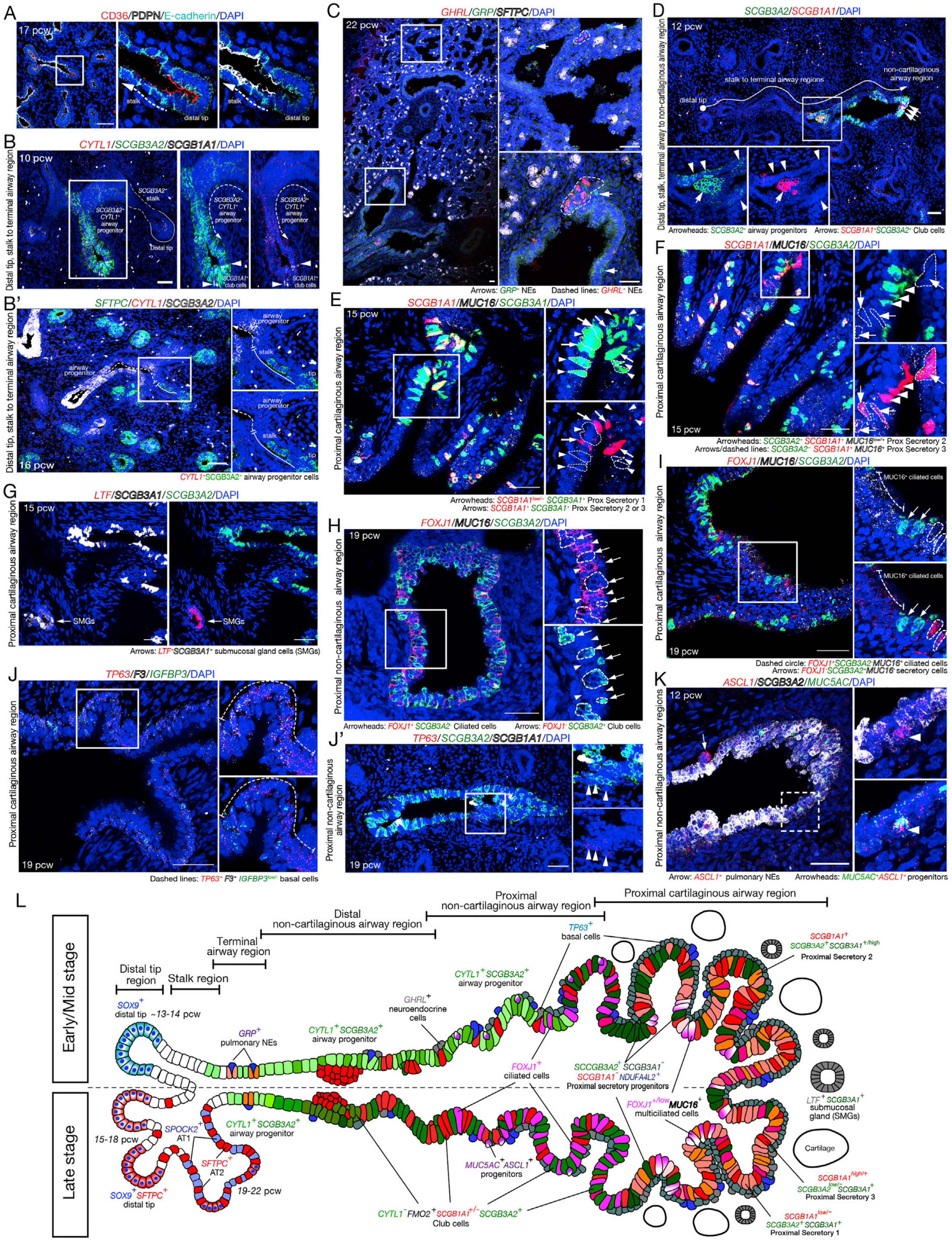

(legend on next page)

Figure S3. Spatial analysis of airway epithelial cells in the developing human lungs by *in situ* HCR, related to Figure 2

(A) Tip and stalk epithelial cells in distal regions of fetal lungs at 17 pcw, immunostained using antibodies against CD36 (tip epithelial cells, red), PDPN (stalk epithelial cells, white), and E-cadherin (epithelium, cyan).

(B and B') Airway progenitor cells in distal fetal lungs at 10 (B) and 16 (B') pcw. The airway progenitor cells marked by *SOX9*$^{-}$/*CYTL1*$^{+}$/*SCGB3A2*$^{+}$ are located proximally to the *CYTL1*$^{-}$/*SCGB3A2*$^{-}$ stalk. *SCGB1A1* indicates club cells (B, white). *SFTPC* is mainly expressed in the tip and partly located in stalk regions (B', green).

(C) GHRL^{+} neuroendocrine (dashed line, red) and GRP^{+} pulmonary neuroendocrine cells (arrow, green) in fetal lungs at 22 pcw. *SFTPC* indicates tip epithelial cells (white).

(D) Airway progenitor (arrowhead) and club cells (arrow) in non-cartilaginous airway regions of fetal lungs at 12 pcw are marked by *SCGB3A2*$^{+}$/*SCGB1A1*$^{-}$ and *SCGB3A2*$^{+}$/*SCGB1A1*$^{+}$, respectively. Tip, stalk, airway progenitor, and club cells are localized progressively more proximally from the distal tip regions to the proximal non-cartilaginous airway regions. *SCGB3A2* (green), *SCGB1A1* (red).

(E) Proximal secretory 1 (arrowhead) and 2 (arrow) are distinguishable by the presence or absence of *SCGB1A1* expression, each marked by *SCGB3A1*$^{+}$/*SCGB1A1*$^{low/-}$/MUC16$^{low/-}$ and *SCGB3A1*$^{+}$/*SCGB1A1*$^{+}$/MUC16$^{low/+}$, respectively, in the proximal cartilaginous airway in 15 pcw fetal lungs. *MUC16*$^{+}$ only cells are MUC16^{+} ciliated cells. *SCGB3A2* (green), *SCGB1A1* (red), *MUC16* (white).

(F) Proximal secretory 2 (arrowhead) and 3 (arrow) are distinguishable by the presence or absence of *SCGB3A2* and *MUC16* expression, marked by *SCGB3A2*$^{+}$/*SCGB1A1*$^{+}$/MUC16$^{low/+}$ and *SCGB3A2*$^{low/-}$/*SCGB1A1*$^{+}$/MUC16^{+}, respectively, in the proximal cartilaginous airway of fetal lungs at 15 pcw. *SCGB3A2* (green), *SCGB1A1* (red), *MUC16* (white).

(G) Submucosal gland cells (arrow) located in SMGs are marked by strong *LTF* expression with *SCGB3A1*$^{+}$/*SCGB3A2*$^{-}$ in the proximal cartilaginous airway regions of fetal lungs at 15 pcw. *SCGB3A2* (green), *LTF* (red), *SCGB3A1* (white).

(H) Ciliated cells and secretory cells are distinguishable by expression of *FOXJ1* (red) or *SCGB3A2* (green) in the non-cartilaginous airway regions at 19 pcw lungs. Ciliated cells (arrowhead), *FOXJ1*$^{+}$/*SCGB3A2*$^{-}$; secretory cells (arrow), *FOXJ1*$^{-}$/*SCGB3A2*$^{+}$.

(I) MUC16^{+} ciliated cells (dashed line), ciliated cells (dashed circle), and secretory cells (arrow) located in the proximal cartilaginous airway regions of fetal lungs at 19 pcw. The MUC16^{+} ciliated cells express *MUC16* (white) with a weak level of *FOXJ1* (red), whereas the ciliated cells only express strong *FOXJ1* without *MUC16* expression. *SCGB3A2* (green).

(J and J') Proximal basal cells (J, dashed line) line the basal layer of the proximal cartilaginous pseudostratified airway in fetal lungs at 19 pcw and are marked by *TP63* (red), *F3* (white), and *IGFBP3* (green). In contrast, only a few *TP63*$^{+}$ basal cells (J', red, arrowheads) are observed in the non-cartilaginous, non-pseudostratified airway regions.

(K) *ASCL1*$^{+}$ pulmonary neuroendocrine (arrow) and MUC5AC^{+}/ASCL1^{+} progenitors (arrowhead) in the non-cartilaginous airway regions of fetal lung at 12 pcw. MUC5AC (green), *ASCL1* (red), *SCGB3A2* (white). DAPI, nuclei. Scale bars, 50 μm.

(L) Diagram describing spatial location of epithelial cell types observed in the developing human lungs.

CellPress
OPEN ACCESS

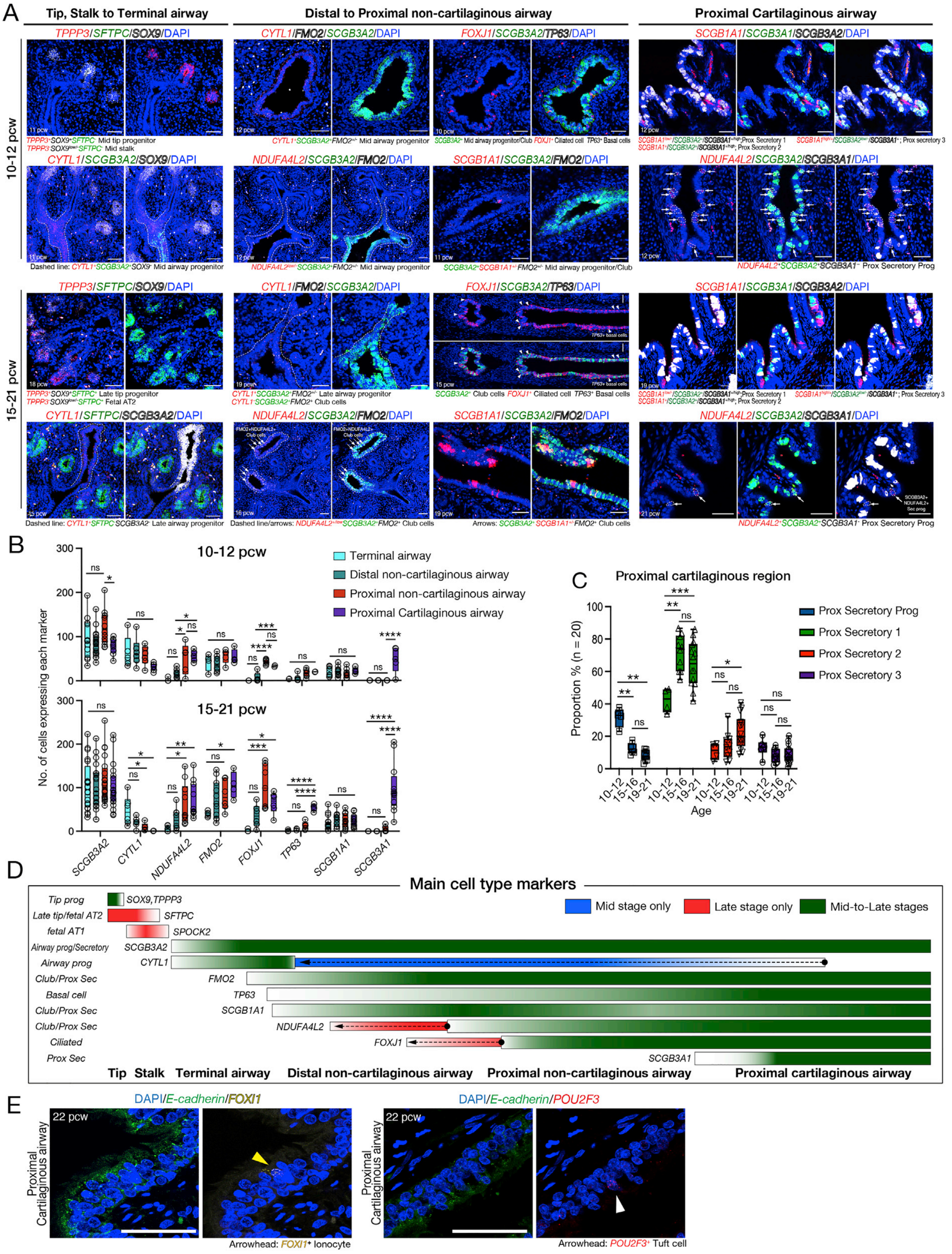

(legend on next page)

Figure S4. Spatiotemporal location, distribution, and quantification of major epithelial cell types along the distal-to-proximal axis of the developing lungs, related to Figure 2

(A) *In situ* HCR analysis of fetal human lung tissues at mid (10–12 pcw) and late (15–21 pcw) stages, showing spatiotemporal location and distribution of major epithelial cell types along the distal to proximal axis of the developing lungs. The lung regions were divided for imaging into tip, stalk to terminal airway, distal to proximal non-cartilaginous airway, and proximal cartilaginous airway.

(B) Quantification of cells expressing marker genes of airway lineages along the airway regions at mid (10–12 pcw, upper) and late (15–21 pcw, lower) stages. *SCGB3A2*, airway progenitors/all secretory lineage cells; *CYTL1*, airway progenitor cells; *NDUFA4L2*, club/proximal secretory cells; *FMO2*, club/proximal secretory cells; *FOXJ1*, ciliated cells; *TP63*, basal cells; *SCGB1A1*, club/proximal secretory cells; *SCGB3A1*, proximal secretory cell subtypes 1–3. Significance was evaluated by 1-way ANOVA with Tukey multiple comparison post-test; n = 3 biological replicates; ns: not significant, $^*p < 0.05$, $^{**}p < 0.01$, $^{***}p < 0.001$, $^{****}p < 0.0001$.

(C) Proportion of proximal secretory progenitor cells, proximal secretory cell subtypes 1–3 within the proximal cartilaginous airway regions by ages, at 10–12, 15–16, and 19–21 pcw. The secretory cells in the proximal cartilaginous airway regions were counted: Prox Secretory Prog, *SCGB3A2*$^+$*SCGB3A1*$^-$*SCGB1A1*$^-$; Prox Secretory 1, *SCGB3A2*$^+$*SCGB3A1*$^+$*SCGB1A1*$^-$; Prox Secretory 2, *SCGB3A2*$^+$*SCGB3A1*$^+$*SCGB1A1*$^+$; Prox Secretory 3, *SCGB3A2*$^-$*SCGB3A1*$^+$*SCGB1A1*$^+$. Club cells located in the non-cartilaginous airway regions were excluded. Significance was evaluated by 2-way ANOVA with Tukey multiple comparison post-test; n = 4 biological replicates; ns: not significant, $^*p < 0.05$, $^{**}p < 0.01$, $^{***}p < 0.001$.

(D) Diagram describing spatiotemporal distribution of major cell-type markers along the distal to proximal axis of the developing lungs, at mid and late stages. Mid stage only, blue; Late stage only, red; Mid-to-late stages, green. Arrows indicate narrowed (*CYTL1*) or expanded (*NDUFA4L2*, *FOXJ1*) distribution after mid- to late-stage transition.

(E) *In situ* HCR analysis of rare cell type markers of putative ionocytes (*FOXI1* yellow) and tuft cells (*POU2F3*, red). *E-cadherin*, green. DAPI, nuclei. Scale bar, 50 μm.

CellPress
OPEN ACCESS

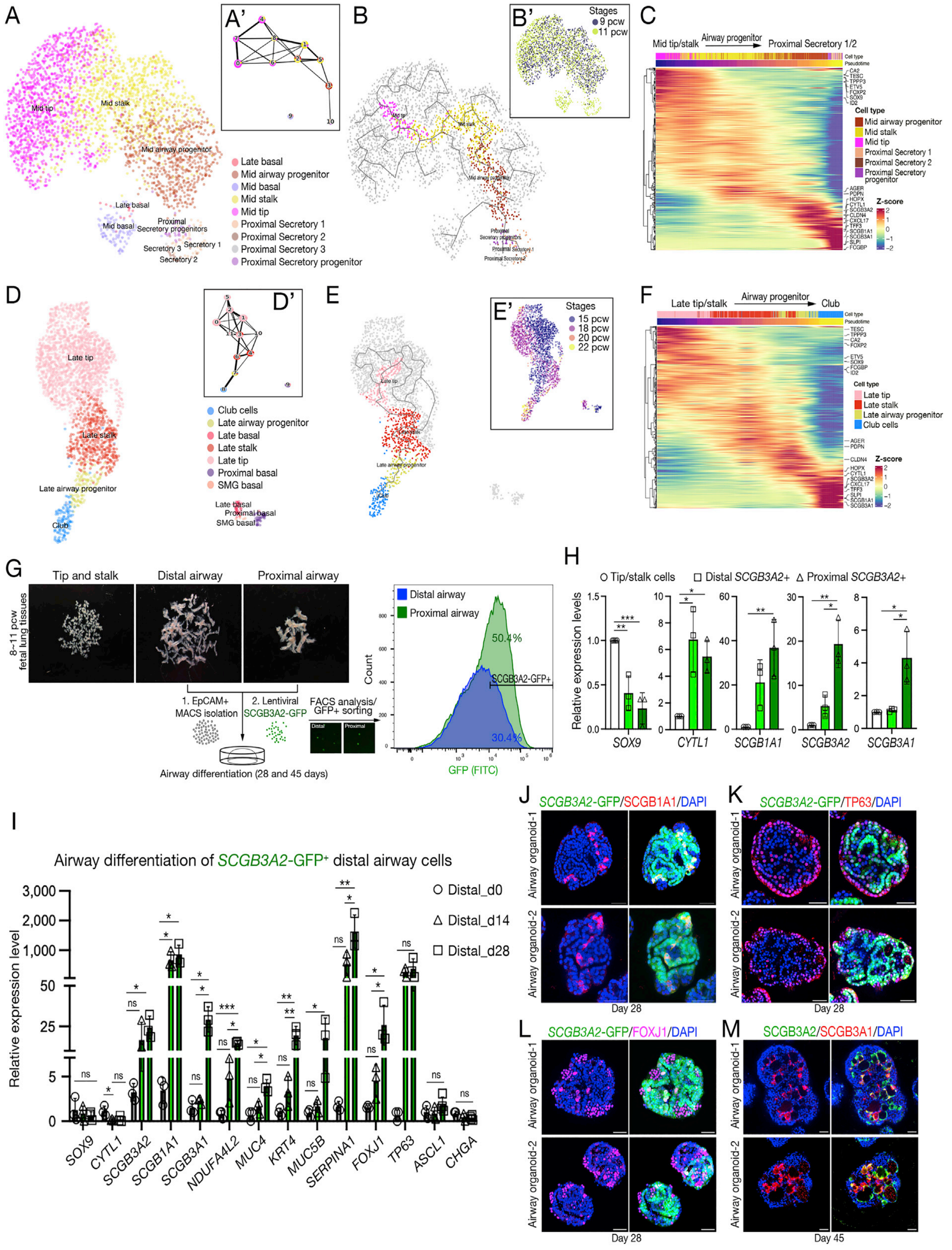

(legend on next page)

Figure S5. Trajectory analysis of airway lineage differentiation via airway progenitor cells in the developing human lung, related to Figure 2

(A and A') UMAP visualization (A) and PAGA analysis (A') of a lineage trajectory from mid-tip to proximal secretory lineage cells, including proximal secretory progenitor and proximal secretory cell subtypes 1 to 3. Mid and late basal cells were shown to be disconnected from other proximal secretory cell types in the PAGA analysis (A').

(B and C) Trajectory UMAPs, by cell type (B) and stages (B'), and the relevant gene expression heatmap (C) displaying the selected lineage trajectory from mid-tip to proximal secretory cell subtypes 1 and 2, analyzed by Monocle 3. (Note that the gray lines in UMAP indicate all of the predicted differentiation paths from a user-defined starting point.). (D, D') UMAP visualization (D) and PAGA analysis (D') of a lineage trajectory from late tip, late stalk, and late airway progenitor, to club cells. Basal cells, including late basal, proximal basal, and SMG basal cells were shown to be left out of the trajectory as they do not connect clearly to the other cell types in this analysis (D').

(E and F) Trajectory UMAPs, by cell types (E) and stages (E'), and the relevant gene expression heatmap (C) showing the selected lineage trajectory from late-tip to club cells, analyzed by Monocle 3.

(G) Purification of distal *SCGB3A2*-GFP^{+} airway cells from human fetal lung tissues at 8–11 pcw. The epithelial cells were isolated using EPCAM magnetic microbeads (MACS) from the dissected distal and proximal airway tissues, followed by infection with lentivirus habouring *SCGB3A2* promoter-driven GFP. The *SCGB3A2*-GFP positive cell fractions were sorted and analyzed by FACS after 48 h and *in vitro* cultured for 28 and 45 days in the airway differentiation medium.

(H) Gene expression profile of the freshly purified *SCGB3A2*-GFP positive cells derived from distal and proximal airway tissues were investigated by qRT-PCR and compared with dissected tip cells. *SOX9*, distal tip progenitor marker. *CYTL1*, airway progenitor marker. *SCGB1A1* and *SCGB3A2*, airway/secretory cell lineage markers. *SCGB3A1*, proximal secretory cell marker. Data was normalized to *SCGB3A2*-GFP negative cells derived from distal tip/stalk tissues; mean ± SD of 3 biological replicates. Significance was evaluated by 1-way ANOVA with Tukey multiple comparison post-test; *$p < 0.05$, **$p < 0.01$, ***$p < 0.001$. (I) Gene expression analysis of the *in vitro* cultured *SCGB3A2*-GFP positive cells (airway progenitors) derived from distal airway tissues by qRT-PCR. Airway organoids were formed from the *SCGB3A2*-GFP positive cells and collected at Day 0, 14, and 28 days after culture for the analysis. Data were normalized to *SCGB3A2*-GFP negative cells derived from distal tip/stalk tissues; mean ± SD of 4 biological replicates. Significance was evaluated by 1-way ANOVA with Tukey multiple comparison post-test; ns: not significant, *$p < 0.05$, **$p < 0.01$. (J-M) Immunofluorescence analysis of two biologically independent, SCGB3A2-GFP+ cell-derived airway organoids cultured in the airway differentiation medium for 28 (J-L) and 45 (M) days. SCGB1A1 (J, red), airway progenitor/secretory cell marker. TP63 (K, red), basal cell marker. FOXJ1 (L, magenta), ciliated cell marker. SCGB3A1 (M, red), proximal secretory cell marker. DAPI, nuclei. Scale bar, 50 μm.

CellPress
OPEN ACCESS

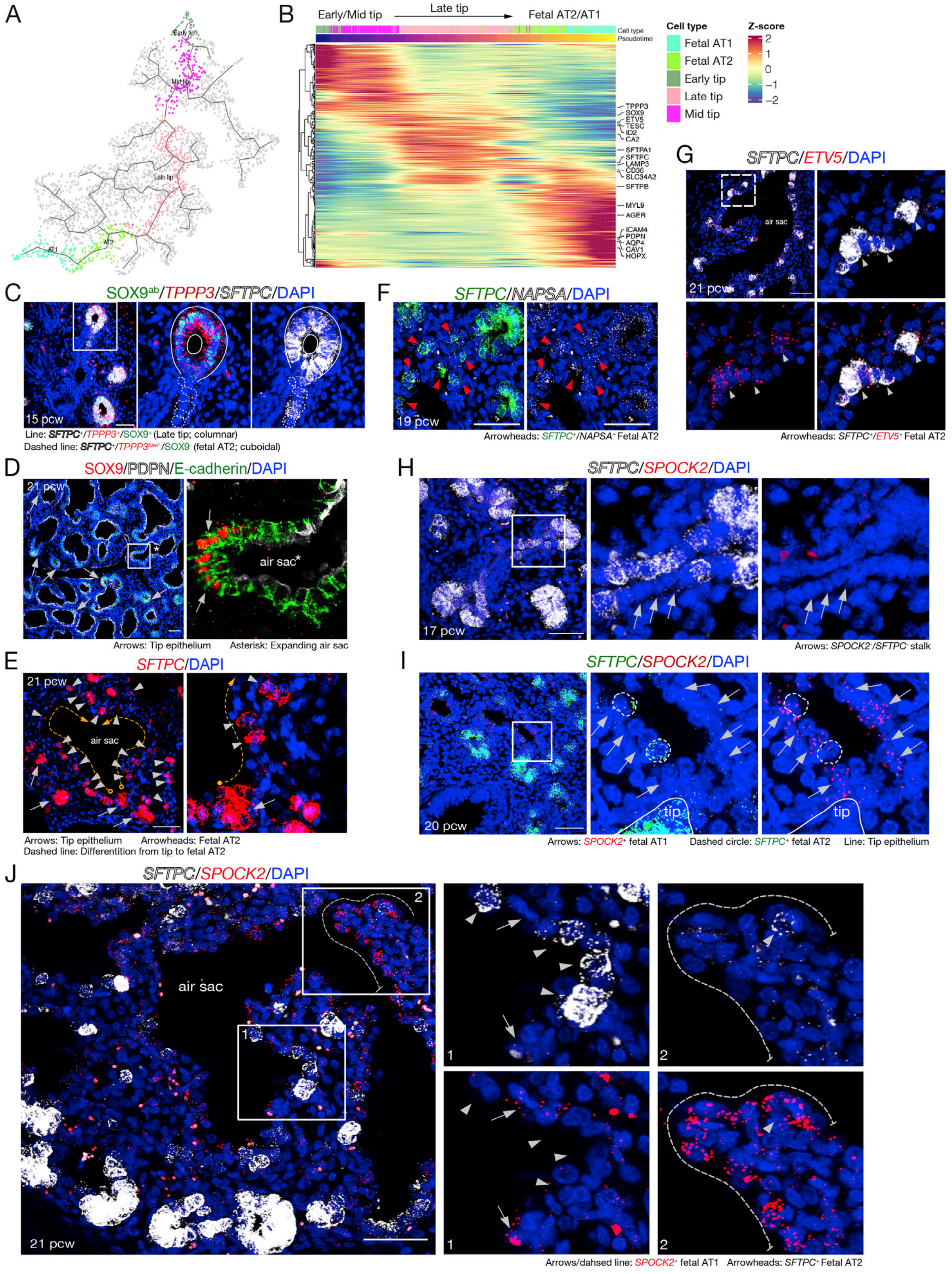

(legend on next page)

Figure S6. Late epithelial tip cells differentiate to AT2 and AT1 cells, related to Figure 3

(A and B) UMAP visualization (A) of a lineage trajectory from early/mid/late tip to fetal AT2 and AT1 cells and the relevant gene expression heatmap (B) showing the selected lineage trajectory analyzed by Monocle 3.

(C) *In situ* HCR (TPPP3 and *SFTPC*) and immunostaining (SOX9) analysis of 15 pcw fetal lung, describing SOX9^{+}*TPPP3*$^{+}$*SFTPC*$^{+}$ tip epithelial progenitors (lines) and SOX9^{-}*TPPP3*$^{-}$*SFTPC*$^{+}$ fetal AT2 cell population (dashed circles) lining the stalk.

(D) Immunostaining of 21 pcw fetal lung using antibodies against SOX9 (red), PDPN (white), and E-cadherin (green). Arrows indicate the late-tip cell population, which does not co-express the stalk marker, PDPN.

(E–G) *In situ* HCR analysis of 19 (F) and 21 pcw (E, G) fetal lungs, showing the *SFTPC*$^{+}$ fetal AT2 cell population (arrowheads) lining the developing air sacs. Arrows indicate *SFTPC*$^{+}$ late-tip cells. (E) *SFTPC* (red). (F, G) NAPSA (white; F) and *ETV5* (red; G) overlap with *SFTPC* in the fetal AT2 cells.

(H–J) *In situ* HCR analysis of distal lung regions at 17 (H), 20 (I), and 21 (J) pcw, visualizing *SFTPC*$^{-}$/*SPOCK2*$^{-}$ stalk cells and *SFTPC*$^{-}$ fetal AT1 cells (arrows). *SFTPC*$^{-}$/*SPOCK2*$^{-}$ stalk cells at 17 pcw (H) began to express *SPOCK2* (red) at 20 pcw (I) and further developed to future AT1 cells (*SFTPC*$^{-}$/*SPOCK2*$^{+}$) at 21 pcw (J). Dashed circles (I) and arrowheads (J) indicate fetal AT2 cells. Dashed line (J) shows fetal AT1 cells lining the developing air sacs. DAPI, nuclei. Scale bars, 50 μm.

CellPress
OPEN ACCESS

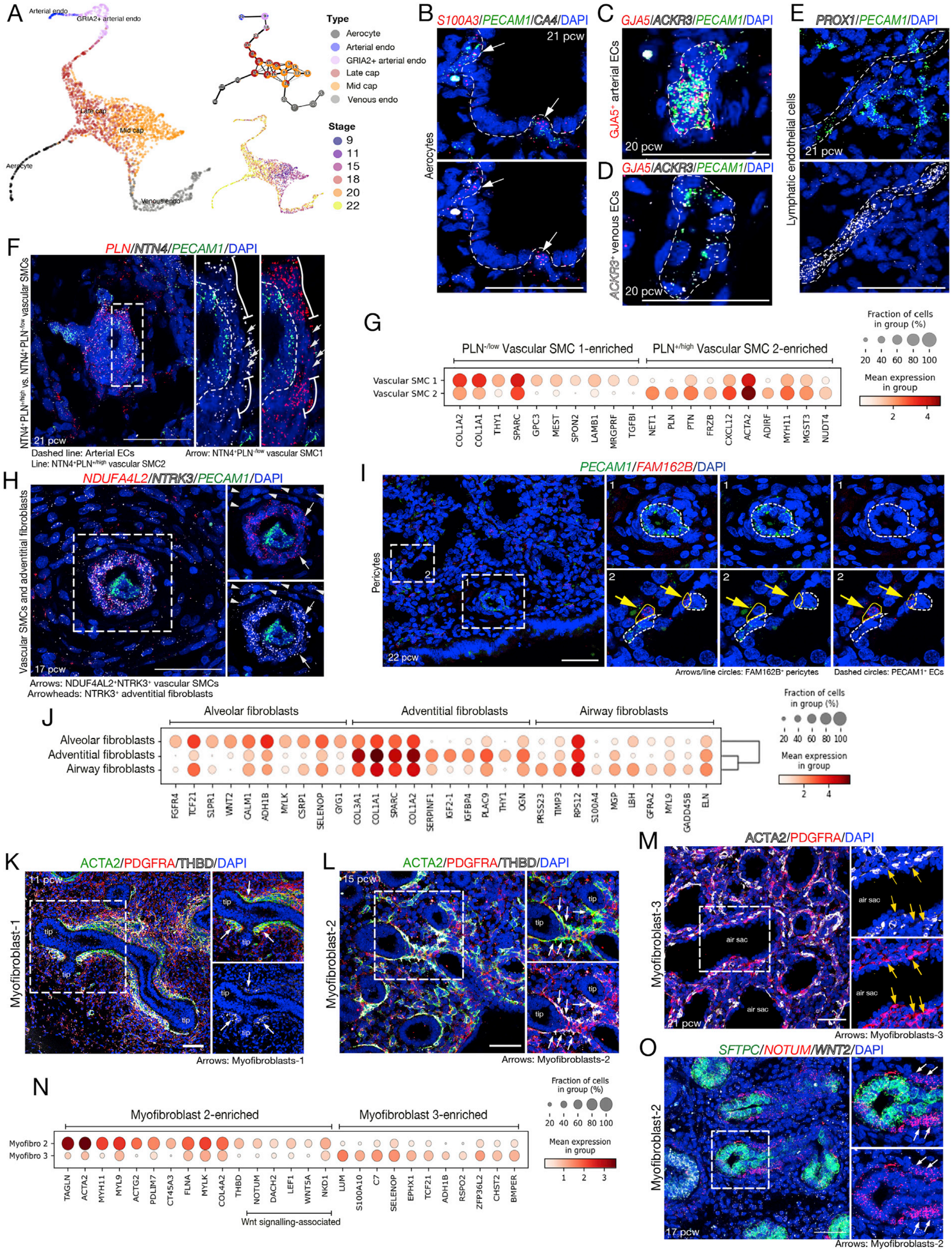

(legend on next page)

Figure S7. Spatial analysis of endothelial and mesenchymal cell types in the developing human lungs by *in situ* HCR assay and immunostaining, related to Figure 4

(A) Trajectory UMAP and PAGA plot visualizing potential endothelial cell lineage hierarchy from Mid/Late capillary endothelial cells to arterial endothelial cells, aerocytes, or venous endothelial cells colored by cell types and stages.

(B–E) *In situ* HCR analysis of distal lung regions at 20 (C, D), and 21 (B, E) pcw.

(B) Aerocytes (*S100A3*$^{+}$ red/*CA4*$^{+}$ white), capillary endothelium (*CA4*$^{+}$ white), and all endothelial cells (*PECAM*$^{+}$, green).

(C) Arterial endothelial cells (*GJA5*$^{+}$ red), (D) venous endothelial cells (*ACKR3*$^{+}$ white), and all endothelial cells (*PECAM*$^{+}$, green).

(E) Lymphatic endothelial cells (*PROX1*$^{+}$ white) and all endothelial cells (*PECAM*$^{+}$, green). DAPI, nuclei. Scale bars, 50 μm.

(F) Vascular SMC 1 and 2 are surrounding arterial endothelial cells (*PECAM1*$^{+}$, dashed line), each marked by *NTN4*$^{+}$/*PLN*$^{-/low}$ (vSMC 1, arrows) and *NTN4*$^{+}$/*PLN*$^{+/high}$ (vSMC 2, lines).

(G) Dot plot describing differential gene expression between vascular SMC 1 and 2.

(H) Vascular SMCs and adventitial fibroblasts in 17 pcw fetal lung. *NDUF4AL2*$^{+}$ red/*NTRK3*$^{+}$ vSMCs (arrows) are surrounded by *NDUF4AL2*$^{-}$/*NTRK3*$^{+}$ adventitial fibroblasts (arrowheads). *PECAM1* (green) indicates an endothelial cell tube.

(I) *FAM162B*$^{+}$ pericytes (red) are surrounding *PECAM1*$^{+}$ endothelial cells (green) in the microvascular regions.

(J) Dot plot describing differential marker gene expression level between alveolar, adventitial and airway fibroblasts.

(K–M) Immunostaining of fetal lung tissues at 11 (K), 15 (L), and 21 (M) pcw, to visualize myofibroblast populations: Myofibroblast-1 (K) and −2 (L) surrounding the developing stalk epithelial tubes, and Myofibroblast-3 (M) surrounding the developing air sacs. ACTA2^{+}/PDGFRA^{+} Myofibroblast-1 (THBD^{weak}; K) and −2 (THBD^{high}, arrows; L). PDGFRA^{+} Myofibroblast-3 at 21 pcw does not express ACTA2 (arrows; M).

(N) Dot plot describing differential gene expression level between myofibroblast-2 and -3. The myofibroblast-2 population showed enriched expression of Wnt signaling associated genes, e.g., *NOTUM*, *LEF1*, and *DACH2*.

(O) *In situ* HCR assay of 17 pcw fetal lung tissues. Myofibroblast-2 expresses *NOTUM* (red), a Wnt antagonist, to block local Wnt signals from alveolar fibroblasts (white, *WNT2*) to the stalk epithelium. DAPI, nuclei. Scale bars, 50 μm.

CellPress
OPEN ACCESS

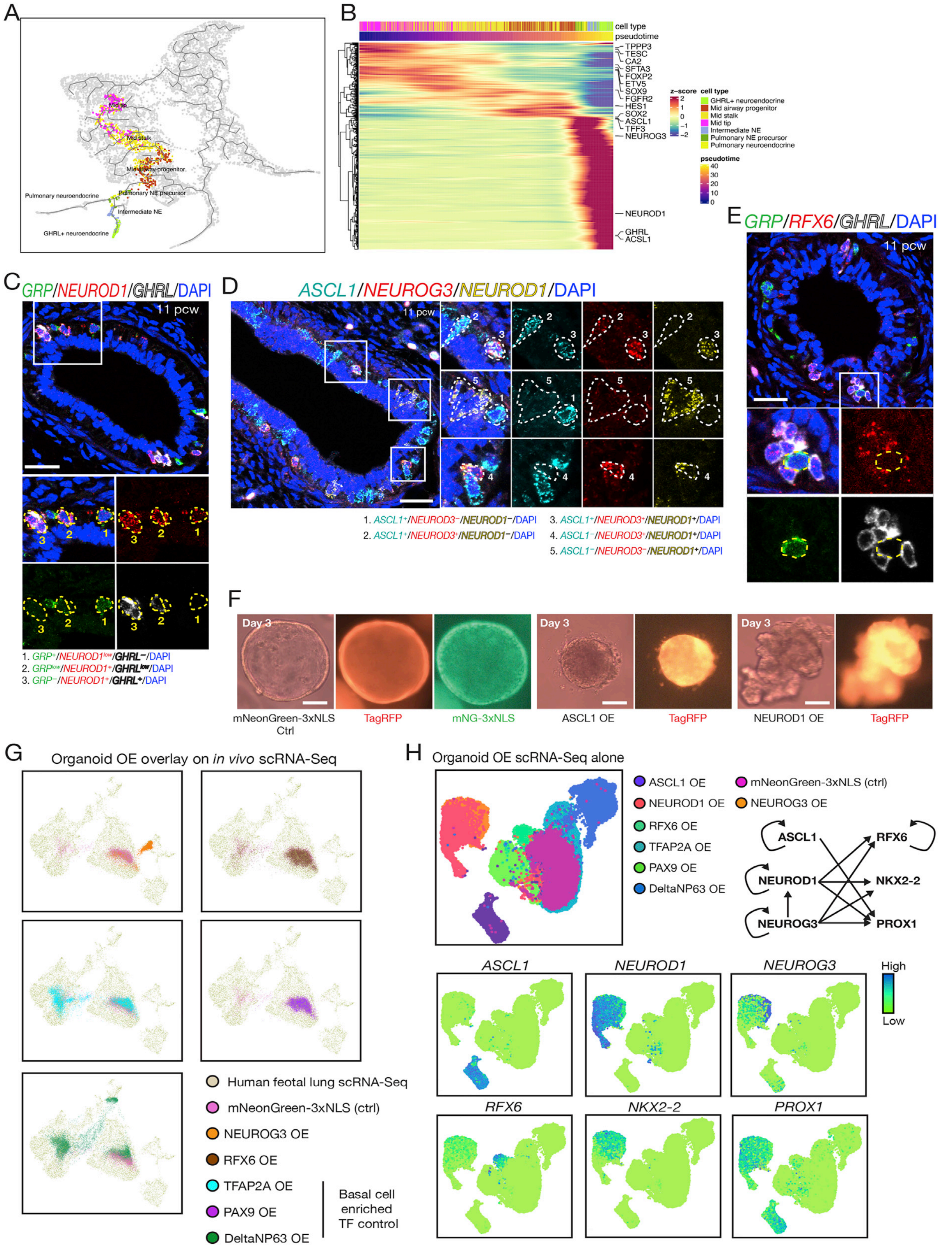

(legend on next page)

Figure S8. Transcription factor regulatory network controlling NE subtypes, related to Figure 7

(A) Selected trajectory from mid-tip cells to *GHRL*$^+$ NE cells via Intermediate NEs, a transition cell population.

(B) Heatmap of genes differentially expressed along the trajectory.

(C) Representative HCR images showing the transition between two types of NE cells. *GRP* (green), *NEUROD1* (red), *GHRL* (white). #1 labeled *GRP*$^+$*NEUROD1*^{low}*GHRL*$^-$ cells, which have just started the transition from GRP$^+$ pulmonary NE/precursor cells. #2 labeled *GRP*^{low}*NEUROD1*$^+$*GHRL*^{low} cells, in transition to *GHRL*$^+$ NE cells. #3 labeled *GRP*$^-$*NEUROD1*$^+$*GHRL*$^+$, *GHRL*$^+$ NE cells. Right: Mean ± SEM of *NEUROD1*$^+$ cell types. 11 pcw: N = 2 fetal lungs, n = 129 *NEUROD1*$^+$ cells; 12 pcw N = 3 fetal lungs, n = 132 *NEUROD1*$^+$ cells. Scale bars, 25 μm in all panels.

(D) Representative HCR images showing *NEUROG3* co-expression with *ASCL1* and *NEUROD1*. Dashed white lines label representative cells showing different combinations of the three transcription factors, further indicated by #1-#5 labeling. *ASCL1* (cyan), *NEUROG3* (red), *NEUROD1* (yellow).

(E) Representative HCR images showing *RFX6* expression in *GHRL*$^+$ NE cells. Dash yellow line labeled *GRP*$^+$RFX6$^-$ pulmonary NE cells. Scale bars, 25 μm in all panels.

(F) Representative epifluorescent microscopic images showing organoid morphology after 3 days of mNeonGreen-3xNLS (control), ASCL1, or NEUROD1 overexpression.

(G) ScRNA-seq results of organoid transcription factor overexpression overlay on human fetal lung scRNA-seq as a reference.

(H) scRNA-seq results of transcription factor overexpression; organoid data only in the UMAP. Selected transcription factor expression was shown in the middle panel. A regulatory network of the selected transcription factors were drawn based on the organoid OE data at the bottom of the panel. (Note that the arrows do not necessarily denote direct interactions).
